# Investigating the Host-Range of the Rust Fungus *Puccinia psidii* sensu lato across Tribes of the Family Myrtaceae Present in Australia

**DOI:** 10.1371/journal.pone.0035434

**Published:** 2012-04-16

**Authors:** Louise Morin, Ruth Aveyard, Jonathan R. Lidbetter, Peter G. Wilson

**Affiliations:** 1 Commonwealth Scientific and Industrial Research Organisation (CSIRO) Ecosystem Sciences, Canberra, Australian Capital Territory, Australia; 2 Central Coast Primary Industries Centre, New South Wales Department of Primary Industries, Gosford, New South Wales, Australia; 3 Royal Botanic Gardens and Domain Trust, Sydney, New South Wales, Australia; Nanjing Agricultural University, China

## Abstract

The exotic rust fungus *Puccinia psidii* sensu lato was first detected in Australia in April 2010. This study aimed to determine the host-range potential of this accession of the rust by testing its pathogenicity on plants of 122 taxa, representative of the 15 tribes of the subfamily Myrtoideae in the family Myrtaceae. Each taxon was tested in two separate trials (unless indicated otherwise) that comprised up to five replicates per taxon and six replicates of a positive control (*Syzygium jambos*). No visible symptoms were observed on the following four taxa in either trial: *Eucalyptus grandis*×*camaldulensis*, *E. moluccana*, *Lophostemon confertus* and *Sannantha angusta*. Only small chlorotic or necrotic flecks without any uredinia (rust fruiting bodies) were observed on inoculated leaves of seven other taxa (*Acca sellowiana*, *Corymbia calophylla* ‘Rosea’, *Lophostemon suaveolens*, *Psidium cattleyanum*, *P. guajava* ‘Hawaiian’ and ‘Indian’, *Syzygium unipunctatum*). Fully-developed uredinia were observed on all replicates across both trials of 28 taxa from 8 tribes belonging to the following 17 genera: *Agonis*, *Austromyrtus*, *Beaufortia*, *Callistemon*, *Calothamnus*, *Chamelaucium*, *Darwinia*, *Eucalyptus*, *Gossia*, *Kunzea*, *Leptospermum*, *Melaleuca*, *Metrosideros, Syzygium*, *Thryptomene*, *Tristania*, *Verticordia*. In contrast, the remaining 83 taxa inoculated, including the majority of *Corymbia* and *Eucalyptus* species, developed a broad range of symptoms, often across the full spectrum, from fully-developed uredinia to no visible symptoms. These results were encouraging as they indicate that some levels of genetic resistance to the rust possibly exist in these taxa. Overall, our results indicated no apparent association between the presence or absence of disease symptoms and the phylogenetic relatedness of taxa. It is most likely that the majority of the thousands of Myrtaceae species found in Australia have the potential to become infected to some degree by the rust, although this wide host range may not be fully realized in the field.

## Introduction

Rust fungi are among the most important plant pathogens worldwide [1]. They can cause severe damage to plants that considerably reduces biomass accumulation and reproduction. For example, rust fungi have caused some of the most destructive diseases of cereal crops, leading to major negative economic and social impacts [2], [3]. With their abundant and typically wind-dispersed spores, rust fungi are also among the most mobile plant pathogens globally [4].

Australian pathologists have feared for many years the possible arrival of the exotic rust fungus *Puccinia psidii* sensu lato (s.l.), commonly known as guava or eucalyptus rust, in Australia [5]–[7]. This plant pathogen is native to South and Central America, but has invaded other regions: Florida [8], California [9] and Hawaii, USA [10], Japan [11] and China [12]. It infects young, actively-growing foliage of plants within the family Myrtaceae, as well as floral buds and young fruits in some hosts [5], [13]. It is known to have a very wide host range within this family of plants, including 129 species from 32 genera in 9 tribes previously reported as susceptible hosts, 86 of which are endemic to Australia [5], [7]–[26]. Of these 129 species, 67 species from 19 genera are field records with the remainder only based on results of experiments performed under controlled conditions. *Puccinia psidii* s.l. has been reported to cause severe damage in some years on *Pimenta dioica* (allspice) in Jamaica [22], *Eucalyptus grandis* [23] and *Psidium guajava* (guava) [24] in Brazil, *Syzygium jambos* (rose apple) in Hawaii [25] and *Melaleuca quinquenervia* in Florida [26], [27]. The implications of such a pathogen incursion in Australia are far-reaching because members of the family Myrtaceae are widespread across the continent and are often major components of natural plant communities [28]. This family includes the major genera *Eucalyptus*, *Corymbia*, *Leptospermum*, *Melaleuca* and *Syzygium* [29], all of which have been previously recorded as hosts for the disease with the exception of *Leptospermum*.

*Puccinia psidii* s.l. was first detected in Australia in the state of New South Wales (NSW) in April 2010 [30], [31]. The origin and pathway of introduction are currently unknown, although a microsatellite analysis is underway to investigate possible origin (M Glen pers. comm.). The rust in Australia was initially identified as *Uredo rangelii* [30], a taxon within the *P. psidii* s.l. complex, based on the presence of a tonsure on urediniospores and absence of teliospores [20]. It was given the common name of myrtle rust. A few months later however, teliospores matching the description of *Puccinia psidii* sensu stricto (s.s.) were observed in the laboratory (L Morin pers. obs.) and in the field [16]. Consequently, Carnegie and Cooper [31] proposed that the name *P. psidii* s.l. was the most appropriate to use until further taxonomic studies of the *Puccinia* and *Uredo* species described on South American Myrtaceae species are undertaken.

The first detection of *P. psidii* s.l. at a property on the NSW Central Coast was on Myrtaceae plants of three different genera: *Agonis flexuosa* ‘Afterdark’, *Syncarpia glomulifera* and *Callistemon viminalis* [30]. Within a few months of this detection, Carnegie and Lidbetter [16] carried out a series of trials to test the susceptibility of key forestry Myrtaceae species, as well as key known hosts of *P. psidii* s.l., to the rust found in Australia. In these trials, the rust infected and completed its life cycle on *Eucalyptus agglomerata*, *E. cloeziana*, *E. grandis*, *E. pilularis* and *M. quinquenervia*. These initial observations and results suggested that the rust newly arrived in Australia was similar to *P. psidii* s.s. and likely to have a wide host range and the potential to become a major threat to natural ecosystems and various plant-based industries.

The aim of this study was to obtain a better understanding of the host-range potential of the Australian accession of *P. psidii* s.l. This information was deemed essential to better target initial surveillance activities and help assess risks to industry and natural ecosystems. An experiment consisting of a series of trials was undertaken under controlled conditions to determine if the rust was capable of infecting and reproducing on a wide range of plant taxa. The experiment included representative plant taxa from each of the 15 tribes of the family Myrtaceae that are present in Australia, based on the most recent molecular phylogeny of the family [29]. An unpublished report on parts of this study can be found in Morin et al. [32].

## Methods

### Plant selection and source

The extensive list of plant taxa included in the experiment was devised in consultation with stakeholders from government agencies and industries (Table S1). The list included 114 representative taxa, including hybrids and cultivars, indigenous to Australia, from the 15 tribes of the subfamily Myrtoideae of the family Myrtaceae [29]. Two *Metrosideros* species indigenous to the Pacific Islands or New Zealand were also included. The list also comprised six additional plant taxa within this subfamily that are not indigenous to Australia and the Pacific region, but have been previously tested with other accessions of *P. psidii* s.l. The nomenclature of the Australian Plant Census [33] was followed for the Australian species names. Govaerts et al. [34] was used as the source for all other species names. For the names of hybrids and cultivars, the international naming conventions were followed. Authorship for plant names is presented in Table S1 or in the text for taxa not included in the table.

Plants were mainly sourced from commercial nurseries or forestry industry contacts, re-potted in fresh potting medium if required (5∶1∶1∶3 straw-based compost, peat moss, river sand, perlite, with 1.4 kg slow-release fertilizer m^−3^ [Aboska®, N∶P∶K 15.16∶6.93∶5.19]), maintained in a glasshouse (16–26°C; natural light and, if required, additional lighting with metal halide lights to maintain a 12-h photoperiod) and fertilized fortnightly with liquid fertilizer (HealthyEarth®, N∶P∶K 18.5∶4.3∶14.5). In some instances, plants were grown from seeds. A representative voucher specimen of each of the taxa, preferentially flowering and/or fruiting, was examined to confirm identification and lodged in the herbarium at the National Herbarium of NSW, Royal Botanic Gardens Sydney (Table S1).

### Rust culture establishment

Since the rust was still highly restricted in distribution in Australia at the time of the study, all experimental work was performed in a high security quarantine facility (QC3) with in-built features including negative pressure and high-efficiency particulate air filters, to ensure containment of rust spores. Foliage of *A. flexuosa* ‘Afterdark’ infected with *P. psidii* s.l. was collected in June 2010 at a property on the Somersby Plateau, NSW Central Coast (Infected Premises no. 1) [30]. Urediniospores were shaken from leaves, suspended in deionized water and sprayed onto healthy, actively-growing plants of *A. flexuosa* ‘Afterdark’ using a hand-held, air-propelled spray atomizer (Model airbrush H-set, Paasche, USA). Each plant was then misted with water, covered with a plastic bag sealed around the pot and placed in a controlled-environment room at 20°C for approx. 18 h in the dark followed by 6 h under fluorescent lights. After 24 h the plastic bags were removed and plants left on the bench in the controlled-environment room (12-h photoperiod, fluorescent lights) until uredinia (fruiting bodies) had fully developed.

A single-uredinium isolate of the rust was then cultured for use in the experiment. It was generated by removing urediniospores from one large uredinium on an infected plant with a fine camel hair paint brush, and dusting them onto leaves of another healthy *A. flexuosa* ‘Afterdark’ plant. The inoculated plant was incubated as outlined above and kept separate from any other rust-infected material. Urediniospores produced on the plant were used to inoculate additional *A. flexuosa* ‘Afterdark’ plants to establish a prolific culture of the single-uredinium isolate to provide inoculum for the experiment. A voucher specimen of the single-uredinium isolate on *A. flexuosa* ‘Afterdark’ has been lodged in the Plant Pathology Herbarium (DAR) of the NSW Department of Primary Industries, Orange, NSW (DAR81284).

### Pathogenicity experiment

#### Plant inoculation

Each taxon was tested in two separate trials to account for any possible variation in time, except for *Eucalyptus obliqua*, *Lindsayomyrtus racemoides*, *Melaleuca howeana*, *Metrosideros nervulosa*, *M. sclerocarpa*, *Osbornia octodonta* and *Syzygium fullagarii*, which were only tested once because of lack of available material. Plant taxa were chosen for each trial based on the presence of new, young growth, since the rust does not infect older foliage. Twenty-three trials consisting of up to 18 taxa each, including the positive control *S. jambos*, were performed.

Inoculum of the single-uredinium isolate was produced on *S. jambos* plants. *Syzygium jambos*, instead of *A. flexuosa* ‘Afterdark’, was selected to mass-produce inoculum and as the control taxon in the experiment because large quantities of plants were more easily accessible. Plants were inoculated and incubated as described below. At three weeks after inoculation, urediniospores were harvested by gently shaking infected foliage above a large piece of foil and then suspended in deionized water with 0.05% Tween 80 (Sigma®). The density of the suspension was determined using a haemocytometer and adjusted to 5×10^4^ urediniospores ml^−1^ with deionized water-Tween 80 solution. The suspension was sprayed onto young shoots of actively-growing plants (five plant replicates per taxa unless indicated otherwise) using a spray atomizer (same as above). Six *S. jambos* plants with young shoots and leaves were inoculated as a positive control in each trial of the experiment.

Inoculated plants were misted with water and placed in humid chambers (40×30×29 cm plastic boxes containing water to a depth of 3 cm and placed in sealed large plastic bags) in a controlled-environment room at 20°C for approx. 18 h in the dark followed by 6 h under fluorescent lights. Plants were then removed from the humid chambers, placed on the bench of the room and maintained at 20°C under a 12-h photoperiod regime.

#### Urediniospore germination assessment

The viability of urediniospores used in each trial was assessed by applying a suspension of spores in liquid paraffin oil (Gold Cross, Biotech Pharmaceuticals) [35] with a fine camel hair paint brush onto the surface of two 1 cm^2^ blocks (approx. 0.5 cm thick) of 2% water agar placed on a microscope slide, with the aid of a dissecting microscope. The density of the urediniospore suspension was adjusted prior to application by dilution with oil to avoid clumping and ensure spore separation on the agar. The slide was placed in a Petri dish lined with moist filter paper and incubated under the same conditions as for inoculated plants. After 24 h, each agar block was placed on a drop of blue-lacto-glycerol stain (0.02 g aniline blue, 10 ml glycerol, 10 ml lactic acid, 10 ml deionized water) to stop the germination process without displacing the spores. Percentage germination was assessed using a light microscope.

#### Disease symptom assessment

**Figure 1:**
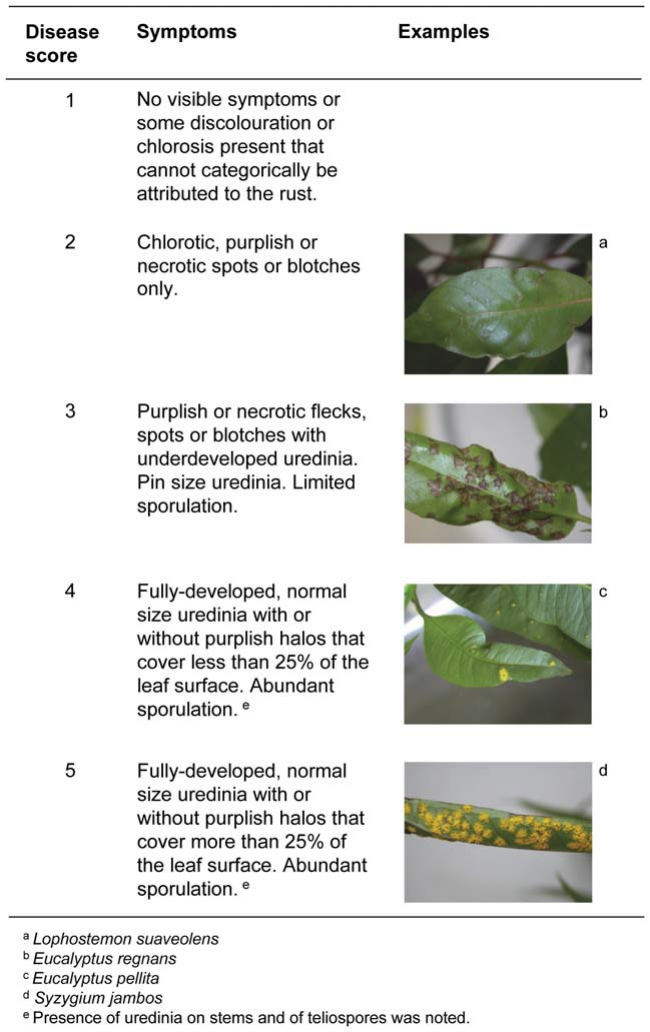
Scoring system for disease symptoms. Scoring system used to categorize visible symptoms observed on leaves three weeks after inoculation of plants with *Puccinia psidii* s.l. (ex Australia, DAR81284). The three leaves with the most severe symptoms on a plant were scored and the highest individual leaf score per plant was assigned to that replicate.

Three weeks after inoculation, all plants were examined individually for disease symptoms. The three leaves with the most severe symptoms on a plant were scored according to the scale in Figure 1 and the highest individual leaf score per plant was assigned to that replicate. The presence of uredinia on stems and of teliospores (another stage of the rust's life-cycle) was also noted. A representative voucher specimen of each taxon with disease symptoms was lodged at the Plant Pathology Herbarium of the NSW Department of Primary Industries (Table S1).

## Results

Germination tests on agar confirmed that urediniospores used in trials were viable, with an average germination of 27% (data not shown). Fully-developed uredinia (disease scores 4 or 5; Fig. 1) were observed on at least one replicate of *S. jambos* in all except one of the trials of the experiment (Table S1). The latter trial was not discarded because many fully-developed uredinia (disease score 5) were observed on at least four replicates of eight of the taxa tested. A summary of results is presented in Table 1 and full detail presented in Table S1. In addition, photographs of the most developed uredinia produced on the various taxa are presented in Figure S1.

**Table 1:** Plant taxa grouped according to disease symptoms at three weeks after inoculation with *Puccinia psidii* s.l. (ex Australia, DAR81284).

Disease symptom^a^	Taxa^b^
No visible symptoms or only chlorotic, purplish or necrotic spots or blotches across all replicates (scores 1 & 2)	*Acca sellowiana* (12); *Corymbia calophylla* ‘Rosea’ (13); *Eucalyptus grandis*×*camaldulensis* (13); *Eucalyptus moluccana* (13); *Lophostemon confertus* (4); *Lophostemon suaveolens* (4); *Psidium cattleyanum* (12); *Psidium guajava* L. ‘Hawaiian’ (12); *Psidium guajava* L. ‘Indian’ (12); *Sannantha angusta* (17); *Syzygium unipunctatum* (11).
Fully-developed uredinia across all replicates (scores 4 & 5)	*Agonis flexuosa* (wild accession) (16); *Agonis flexuosa* ‘Afterdark’ (16); *Austromyrtus dulcis* (12); *Beaufortia schaueri* (6); *Beaufortia sparsa* (6); *Callistemon* ‘Kings Park Special’ (6); *Callistemon viminalis* (6); *Calothamnus quadrifidus* (6); *Chamelaucium uncinatum* (17); *Darwinia citriodora* c (17); *Eucalyptus cloeziana* (13); *Gossia inophloia* c (12); *Kunzea ambigua* hybrid (16); *Kunzea ericoides* (16); *Kunzea pomifera* (16); *Leptospermum continentale* ‘Horizontalis’ (16); *Leptospermum* ‘Lipstick’^c^ (16); *Leptospermum* ‘Rudolph’ (16); *Leptospermum* ‘White Wave’ (16); *Melaleuca alternifolia* (6); *Melaleuca linariifolia* ‘Claret Tops’ (6); *Metrosideros collina* ‘Tahiti’ (9); *Metrosideros excelsa* ‘Golden Dawn’ (9); *Metrosideros sclerocarpa* (9); *Syzygium australe* ‘Meridian Midget’ (11); *Thryptomene calycina* (17); *Tristania neriifolia* (10); *Verticordia plumosa* (17)
Range of symptoms across replicates (scores 1–5)	Control: *Syzygium jambos* c (11); *Allosyncarpia ternata* (13); *Angophora costata* (13); *Angophora floribunda* (13); *Asteromyrtus magnifica* (16); *Backhousia citriodora* (8); *Backhousia myrtifolia* (8); *Callistemon* ‘Hannah Ray’ (6); *Callistemon citrinus* ‘White Anzac’ (6); *Callistemon linearifolius* (6); *Corymbia citriodora* accessions no. 1 and 2 (13); *Corymbia ficifolia* (13); *Corymbia gummifera* (13); *Corymbia henryi* (13); *Corymbia intermedia* (13); *Corymbia maculata* (13); *Corymbia tessellaris* (13); *Corymbia torelliana* (13); *Corymbia variegata*×*torelliana* (13); *Decaspermum humile* (12); *Eucalyptus agglomerata* (13); *Eucalyptus argophloia* (13); *Eucalyptus campanulata* (13); *Eucalyptus cladocalyx* (13); *Eucalyptus diversicolor* (13); *Eucalyptus dunnii* (13); *Eucalyptus globulus* subsp. *bicostata* c (13); *Eucalyptus globulus* subsp. *globulus* c (13); *Eucalyptus gomphocephala* (13); *Eucalyptus grandis* (13); *Eucalyptus haemastoma* (13); *Eucalyptus laevopinea* (13); *Eucalyptus longirostrata* (13); *Eucalyptus marginata* subsp. *marginata* (13); *Eucalyptus nitens* c (13); *Eucalyptus obliqua* (13); *Eucalyptus occidentalis* (13); *Eucalyptus olida* (13); *Eucalyptus pellita* (13); *Eucalyptus pilularis* (13); *Eucalyptus populnea* (13); *Eucalyptus punctata* (13); *Eucalyptus regnans* (13); *Eucalyptus resinifera* subsp. *hemilampra* (13); *Eucalyptus saligna* (13); *Eucalyptus siderophloia* (13); *Eucalyptus smithii* (13); *Eucalyptus tereticornis* (13); *Eucalyptus tindaliae* (13); *Eucalyptus wandoo* subsp. *wandoo* (13); *Kunzea baxteri* (16); *Leptospermum* ‘Day Dream’ (16); *Leptospermum* ‘Love Affair’ (16); *Leptospermum* ‘Mesmer Eyes’ (16); *Leptospermum* ‘Pink Cascade’ (16); *Leptospermum* ‘Rhiannon’ (16); *Leptospermum* ‘Riot’ (16); *Leptospermum laevigatum* c (16); *Leptospermum morrisonii* ‘Burgundy’ (16); *Leptospermum polygalifolium* (16); *Leptospermum polygalifolium*×*scoparium* (16); *Leptospermum trinervium* (16); *Lindsayomyrtus racemoides* c (15); *Melaleuca ericifolia* (6); *Melaleuca howeana* (6); *Melaleuca quinquenervia* (6); *Metrosideros nervulosa* (9); *Osbornia octodonta* (5); *Pimenta dioica* (12); *Regelia velutina* (6); *Syncarpia glomulifera* (14); *Syzygium anisatum* (11); *Syzygium australe* ‘Captain Cook’ (11); *Syzygium fibrosum* (11); *Syzygium floribundum* (11); *Syzygium francisii* c (11); *Syzygium fullagarii* (11); *Syzygium luehmannii* c (11); *Syzygium oleosum* (11); *Syzygium smithii* rheophytic form (11); *Tristaniopsis laurina* (7);*Verticordia chrysantha* (17); *Xanthostemon chrysanthus* (3)

a Scores based on scoring system in Figure 1.

b Authorship of names presented in Table S1.Number in parentheses correspond to tribe number based on Wilson et al. [29].

c Teliospores present on one or more replicates.

No visible symptoms were observed on four taxa inoculated with the rust: *Eucalyptus grandis*×*camaldulensis*, *E. moluccana*, *Lophostemon confertus* and *Sannantha angusta* (Tables 1, S1). Many of the replicates of another seven taxa (*Acca sellowiana*, *Corymbia calophylla* ‘Rosea’, *Lophostemon suaveolens*, *Psidium cattleyanum*, *P. guajava* ‘Hawaiian’ and ‘Indian’, *Syzygium unipunctatum*) also did not develop visible symptoms. Small chlorotic or necrotic flecks (disease score 2), however, were observed on inoculated leaves of some replicates of these taxa (Table S1).

Fully-developed uredinia (disease scores 4 or 5) were observed on all replicates of 28 taxa across both trials (Tables 1, S1, Fig. S1). In contrast, a range of symptoms from fully-developed uredinia (disease scores 4 or 5) to underdeveloped uredinia (disease score 3), chlorotic or necrotic flecks (disease score 2) or no visible symptoms (disease score 1) was observed on the replicates of the remaining 83 taxa inoculated (Tables 1, S1, Fig. S1). Across all plant taxa tested we observed that the rust only infected actively-growing foliage. The rust did not develop uredinia on older foliage, although in some instances necrotic flecks or spots were observed. The presence of teliospores was microscopically confirmed on *Darwinia citriodora*, *Eucalyptus globulus* subsp. *bicostata* and *globulus*, *E. nitens, Gossia inophloia*, *Leptospermum* ‘Lipstick’, *L. laevigatum*, *Lindsayomyrtus racemoides*, *Syzygium francisii*, *S. jambos* and *S. luehmannii*.

## Discussion

While many rust fungi have a host range restricted to a single plant species, there are some, such as *Puccinia coronata* (crown rust of grasses) [36], *Phakopsora pachyrhizi* (soybean rust) [37] and *Uromyces striatus* (alfalfa rust) [38], that infect a wide range of different plant species within a family. *Puccinia psidii* s.l. falls in the latter group as it infects species from many different genera within the family Myrtaceae [5], [7]–[26]. All hosts recorded in previous studies were from the subfamily Myrtoideae, except for *Heteropyxis natalensis* Harv. [14], which is now considered a member of the subfamily Psiloxyloideae in the Myrtaceae [29]. In the early 2000 s, pathogenicity tests conducted with a Brazilian accession of *P. psidii* s.l. (ex Itapetininga) showed that 58 of the 67 Australian Myrtaceae species, from all of the 8 different tribes tested except Lophostemoneae, were susceptible to some degree to the rust [21]. In our study using an Australian accession of *P. psidii* s.l., we showed that it is capable of infecting and developing uredinia on 111 of the 122 taxa inoculated, belonging to all tribes, except again the Lophostemoneae, in the subfamily Myrtoideae of the Myrtaceae.

Teliospores were observed on some plants of 11 different taxa used in our experiment. In the field in the native range, teliospores have been reported to occur more frequently on some hosts such as *S. jambos* and during warmer months [39]. Teliospores are also reported to be as common as urediniospores in the field in Hawaii [17]. In previous experimental work, teliospores production was stimulated by incubation of infected hosts at temperatures higher than 20°C but lower than 30°C [40], [41], [42]. Considering that environmental conditions during each of the trials of our experiment were the same, we can only speculate that teliospore production on a few taxa was the result of an interaction between some plants and growing conditions in the glasshouse before the experiment.

It is important to reiterate that the disease scores recorded for individual plants of each taxon in our experiment are not a measure of the overall severity of the rust on plants, but rather a qualitative assessment of the type of symptoms that developed. For example, while all replicates of *Calothamnus quadrifidus* were given a disease score of 4 or 5 because fully-developed uredinia were observed (Tables 1, S1, Fig. S1), only a few leaves in each plant were actually infected by the rust. Measuring the impact of *P. psidii* s.l. on the growth and reproduction of susceptible plants is better performed in the field where plants are exposed to natural, fluctuating conditions that influence their phenotype, particularly their growth rate and hence availability of young foliage suitable for rust infection.

While testing of more replicate plants per taxon could have increased the chances of finding individuals susceptible to the rust, it was not logistically feasible within the timeframe of the study and the limited space in the quarantine facility. Increasing the number of replicate plants would have been especially relevant for those plant accessions that originated from a bulk collection of seed from a large number of individual trees in the wild and therefore likely to be highly variable (e.g. *Eucalyptus smithii*). Further, inclusion of additional accessions of the various taxa tested in our experiment could have produced different results depending on the level of variation that exists between populations. This additional testing would be particularly relevant for taxa that did not develop uredinia in our experiment to increase confidence in defining these taxa as immune or resistant to the rust. For example, Zauza et al. [21] observed that while none of the plants of some seedlots of *Eucalyptus amplifolia* Naudin subsp. *amplifolia*, *E. brassiana* S.T.Blake, *E. diversicolor*, *E. pellita*, *E. resinifera*, *E. tereticornis* and *Syzygium australe* inoculated with *P. psidii* s.l. (ex. Itapetininga, Brazil) developed any disease symptoms, other accessions of the same species comprised some individuals that were assessed as susceptible.

The range of symptoms observed on 83 of the taxa tested was encouraging as it indicates the possible existence of some levels of genetic resistance in these taxa. However, the range of symptoms observed on these taxa cannot be categorically attributed to the genetic make-up of the individuals tested. For example, the variation in symptoms development observed on the *Leptospermum* hybrid cultivars ‘Day Dream’, ‘Love Affair’, ‘Mesmer Eyes’ ‘Pink Cascade’, ‘Rhiannon’ and ‘Riot’ was surprising considering that these taxa are of hybrid origin and propagated clonally. While we carefully selected plants that had young growth at the time of inoculation, we suspect that the rate at which the foliage was produced may have affected susceptibility to infection by the rust. Such a phenomenon has been observed with the rust fungus *Phragmidium violaceum* on blackberry (*Rubus fruticosus* L. aggregate) [43]. Young plants grown under uniform conditions should be used to screen for resistant genotypes to the rust in order to limit the influence of plant phenotype (especially with regards to growth rate) on results.

Overall, there was no apparent association in our study between the presence or absence of disease symptoms and the phylogenetic relatedness of taxa within the family. In other words, development of the disease in one species in a tribe did not mean that a related species or genus also developed the disease. This was not the case, however, for accessions of the two species tested within the Lophostemoneae tribe (*L. confertus* and *L. suaveolens*), which did not develop any rust infection following inoculation in either trial. Only signs of a resistance reaction in the form of small necrotic spots were observed on young leaves of the accession of *L. suaveolens* used in our experiment. *Lophostemon confertus* was also found to be immune to another *P. psidii* s.l. accession in a previous study [21]. It is noteworthy that the rust has recently been observed on *L. suaveolens* in the field in northern NSW (JR Lidbetter, pers. obs. Nov. 2011). This highlights that variation in resistance to the rust exists between accessions of this species.

Our study revealed several differences between the host range of the Australian accession of *P. psidii* s.l. and two others from Florida [19] and Brazil [21] (Table 2). Of most interest was the inability of the Australian rust accession to develop uredinia on *P. guajava* compared to the accession from Florida [19]. In contrast, the Australian rust accession developed uredinia on five plant taxa (*Austromyrtus dulcis*, *Corymbia tessellaris*, *Melaleuca ericifolia*, *S. australe*, *S. jambos*), which were found to be resistant to the other two accessions. Physiological specialization within *P. psidii* s.l. has been demonstrated in cross-inoculation experiments [22], [39], [44]. For example, Coelho et al. [44] identified three groups of rust biotypes, each pathogenic on different host combinations: *P. guajava* only, *E. grandis* and *P. guajava* or *E. grandis* and *S. jambos*. The development of a differential set of clonal host lines would enable a more accurate determination of the biotypes that exist within *P. psidii* s.l.

**Table 2:** Comparison of results of pathogenicity testing performed under controlled conditions with *Puccinia psidii* s.l. from Australia (DAR81284) (based on this study) and other accessions of the rust from Florida [19] and Brazil [21].

Taxa^a^	Presence of uredinia^b^		
	Origin of rust accession		
	Australia	Florida	Brazil^c^
*Acca sellowiana* d	−	−	×
*Asteromyrtus dulcis* e	+	×	−
*Corymbia calophylla* ‘Rosea’	−	×	−
*Corymbia tessellaris*	+	×	−
*Eucalyptus grandis*	+	−	+
*Eucalyptus grandis*×*camaldulensis*	−	×	×
*Eucalyptus moluccana*	−	×	+^f^
*Eucalyptus pellita*	+	×	+/−g
*Eucalyptus resinifera* h	+	×	+/−i
*Eucalyptus tereticornis* j	+	−	+/−k
*Lophostemon confertus*	−	−	−
*Lophostemon suaveolens*	−	−	−
*Melaleuca ericifolia*	+	−	−
*Psidium guajava*	−	+^l^/−m	−
*Psidium cattleyanum*	−	−l ^, ^ m	−
*Sannantha angusta*	−	−	−
*Syzygium australe*	+	−	−n
*Syzygium jambos*	+	−l ^, ^ m	−
*Syzygium unipunctatum*	−	−	−

a Only taxa that did not develop any uredinia with the Australian accession of the rust or with one of the other two accessions used in other studies are included.

b + = uredinia present; − = uredinia absent; × = taxon not tested.

c Only taxa categorized as 100% resistant are included.

d Synonym of *Feijoa sellowiana*.

e Listed as *Asteromyrtus dulcia* in Zauza et al. [21].

f Only plants of accessions 15877 & 20010 from the CSIRO Australian Tree Seed Centre tested were found to be resistant.

g Only plants of accession 18324 from the CSIRO Australian Tree Seed Centre tested were found to be resistant.

h Identified to subspecies level in current study.

i Only plants of accession 13953 from the CSIRO Australian Tree Seed Centre tested were found to be resistant.

j This species was also found to be susceptible to the rust accession recently found in Japan in pathogenicity testing conducted by Kawanishi et al. [11].

k Only plants of accession 17763 from the CSIRO Australian Tree Seed Centre tested were found to be resistant.

l With rust accession recovered from *Pimenta dioica*.

m With rust accession recovered from *Melaleuca quinquenervia*.

n Relates to accession RF12 from the CSIRO Australian Tree Seed Centre.

At the commencement of this study only three plant species had been recorded as hosts of *P. psidii* s.l. in the field in Australia [30]. In the 21 months since, over 175 plant species have been reported as hosts in the field in the states of NSW [45] and Queensland [46]. This has been by far the fastest accumulation of host records ever recorded for any biotype within the *P. psidii* s.l. complex [16]. Many of the hosts recorded in the field had already been identified as potential hosts as part of this study, only to be confirmed in the field within months. At the time of writing this paper, 56 of the species (and a number of hybrids) that developed uredinia in the experiment performed in this study had not yet been observed to be infected in the field. Some of this discrepancy can be explained by the fact that many of these taxa are not endemic or commonly cultivated in the current range of the rust in Australia. The combination of field observations since the incursion of *P. psidii* s.l. in Australia [45], [46] and results from this study has seen the worldwide host list of *P. psidii* s.l. increased from 129 [16] to over 300. This fact alone emphasizes the risk the Australian flora faces in light of this new introduction, having not co-evolved with *P. psidii* s.l.

The species commonly under cultivation in nurseries and gardens that are so far severely affected by *P. psidii* s.l. in the field in Australia include *A. flexuosa*, *Gossia inophloia, Syzygium anisatum* and *S. jambos* [16], [31]. In the experiment performed in this study, fully-developed uredinia were observed across all replicates of both *A. flexuosa* and *G. inophloia*, while a range of symptoms were recorded for *S. anisatum* and *S. jambos*. A range of symptoms was also observed on *M. quinquenervia*, which has recently been found to be severely damaged by the rust in native bushland (P Entwistle pers. comm.). The latter results may reflect phenotypic and/or genotypic differences between individual plants of each taxon. Other relatively frequent species severely damaged by the rust in native bushland include *Rhodamnia rubescens* (Benth.) Miq. and *Rhodomyrtus psidioides* (G.Don) Benth. [16], which were not part of the test list of this study.

It is most likely that the majority of the thousands of species of Myrtaceae found in Australia have the potential to become infected to some degree by *P. psidii* s.l., although this wide host range may not be fully realized in the field. There are many factors required for the disease to develop in the field, such as the presence of actively-growing young shoots, climatic conditions conducive to infection and availability of abundant inoculum. For example, some of the species found to be susceptible to *P. psidii* s.l. in a controlled-environment experiment performed by Rayachhetry et al. [19] were initially observed not to develop disease symptoms in the field, even when growing near severely infected *M. quinquenervia* trees. A better understanding of the relative roles of phenotypic and genetic resistance on the development of *P. psidii* s.l. epidemics in the field, when conditions are conducive for the disease, would advance our understanding of the dynamics at play here.

## Supporting Information

Table S1 **Number of replicates (plants) of each taxon in each of the five disease score categories (** **Fig. 1** **) at three weeks after inoculation with** ***Puccinia psidii*** **s.l. (ex Australia, DAR81284).**
(DOC)

Figure S1 **Photographs of the most developed uredinia produced on the various taxa.** Photographs of the most developed uredinia produced on the various taxa at three weeks after inoculation with *Puccinia psidii* s.l. (ex Australia, DAR81284).
(PDF)
