# Supplementary material for: Investigating the Host-Range of the Rust Fungus Puccinia psidii sensu lato across Tribes of the Family Myrtaceae Present in Australia
Source: PLoS One. 2012 Apr 16;7(4):e35434. doi: 10.1371/journal.pone.0035434 (PMC3327671; doi:10.1371/journal.pone.0035434)
Supplement: Table S1 — Number of replicates (plants) of each taxon in each of the five disease score categories ( Fig. 1 ) at three weeks after inoculation with Puccinia psidii s.l. (ex Australia, DAR81284). (DOC) [file pone.0035434.s001.doc]

**Table S1. Number of replicates (plants) of each taxon in each of the five disease score categories (Fig. 1) at 3 weeks after inoculation with *Puccinia psidii* s.l. (ex Australia, DAR81284).**

| **Tribe**  **no.** ^a^ | **Plant taxa** ^b^ | **Number of replicates** ^c^ | | | | | **Plant voucher accession no.** ^d^ | **Diseased plant voucher accession no.** ^e^ |
| --- | --- | --- | --- | --- | --- | --- | --- | --- |
|  |  | **Disease score** | | | | |  |  |
|  |  | **1** | **2** | **3** | **4** | **5** |  |  |
|  | **Control** |  |  |  |  |  |  |  |
| 11 | *Syzygium jambos* (L.) Alston ^f, g, h, i^ | 25 | 8 | 38 | 4 | 62 | NSW883962 | DAR81648 |
|  | **Taxa with no visible symptoms or only chlorotic, purplish or necrotic spots or blotches** | | | | | | | |
| 4 | *Lophostemon confertus* (R.Br.) Peter G.Wilson & J.T.Waterh. | 10 | 0 | 0 | 0 | 0 | NSW883937 | — ^j^ |
| 4 | *Lophostemon suaveolens* (Sol. ex Gaertn.) Peter G.Wilson & J.T.Waterh. | 4 | 6 | 0 | 0 | 0 | NSW883938 | DAR81624 |
| 11 | *Syzygium unipunctatum* (B.Hyland) Craven & Biffin ^k^ | 8 | 1 | 0 | 0 | 0 | NSW883966 | DAR81652 |
| 12 | *Acca sellowiana* (O.Berg) Burret ^h, k^ | 6 | 3 | 0 | 0 | 0 | NSW883852 | DAR81541 |
| 12 | *Psidium cattleyanum* Afzel. ex Sabine ^h^ | 6 | 4 | 0 | 0 | 0 | NSW883950 | DAR81636 |
| 12 | *Psidium guajava* L. 'Hawaiian' ^h, k^ | 2 | 7 | 0 | 0 | 0 | NSW883951 | DAR81637 |
| 12 | *Psidium guajava* L.'Indian' ^h, k^ | 4 | 5 | 0 | 0 | 0 | — | DAR81638 |
| 13 | *Corymbia calophylla* (R.Br. ex Lindl.) K.D.Hill & L.A.S.Johnson ‘Rosea’ | 8 | 2 | 0 | 0 | 0 | NSW883871 | DAR81560 |
| 13 | *Eucalyptus grandis* × *camaldulensis* ^k^ | 10 | 0 | 0 | 0 | 0 | NSW883897 | — |
| 13 | *Eucalyptus moluccana* Wall. ex Roxb. | 10 | 0 | 0 | 0 | 0 | NSW883902 | — |
| 17 | *Sannantha angusta* (A.R.Bean) Peter G.Wilson | 10 | 0 | 0 | 0 | 0 | NSW883953 | — |
|  | **Taxa with fully-developed uredinia across all replicates** | | | | | |  |  |
| 6 | *Beaufortia schaueri* Preiss ex Schauer ^g^ | 0 | 0 | 0 | 5 | 5 | NSW883862 | DAR81551 |
| 6 | *Beaufortia sparsa* R.Br. ^g, k^ | 0 | 0 | 0 | 1 | 8 | NSW883863 | DAR81552 |
| 6 | *Callistemon* 'Kings Park Special' ^g, k^ | 0 | 0 | 0 | 2 | 7 | NSW883865 | DAR81554 |
| 6 | *Callistemon viminalis* (Sol. ex Gaertn.) G.Don ^g^ | 0 | 0 | 0 | 0 | 10 | NSW883867 | DAR81556 |
| 6 | *Calothamnus quadrifidus* R.Br. ^g^ | 0 | 0 | 0 | 4 | 6 | NSW883869 | DAR81558 |
| 6 | *Melaleuca alternifolia* (Maiden & Betche) Cheel ^g^ | 0 | 0 | 0 | 0 | 10 | NSW883939 | DAR81625 |
| 6 | *Melaleuca linariifolia* Sm. 'Claret Tops' ^g^ | 0 | 0 | 0 | 1 | 9 | NSW883942 | DAR81628 |
| 9 | *Metrosideros collina* (J.R.Forst. & G.Forst.) A.Gray'Tahiti' ^g, h^ | 0 | 0 | 0 | 1 | 9 | NSW883944 | DAR81630 |
| 9 | *Metrosideros excelsa* Sol. ex Gaertn.'Golden Dawn' ^g, h, l^ | 0 | 0 | 0 | 0 | 10 | NSW883945 | DAR81631 |
| 9 | *Metrosideros sclerocarpa* J.W.Dawson ^g, m^ | 0 | 0 | 0 | 2 | 3 | NSW883947 | DAR81633 |
| 10 | *Tristania neriifolia* (Sims) R.Br. in W.T.Aiton ^g^ | 0 | 0 | 0 | 1 | 9 | NSW883968 | DAR81654 |
| 11 | *Syzygium australe* (J.C.Wendl. ex Link) B.Hyland 'Meridian Midget' ^g^ | 0 | 0 | 0 | 0 | 10 | NSW883957 | DAR81643 |
| 12 | *Austromyrtus dulcis* (C.T.White) L.S.Sm. ^g^ | 0 | 0 | 0 | 0 | 10 | NSW883859 | DAR81548 |
| 12 | *Gossia inophloia* (J.F.Bailey & C.T.White) N.Snow & Guymer ^g, i^ | 0 | 0 | 0 | 2 | 8 | NSW883916 | DAR81604 |
| 13 | *Eucalyptus cloeziana* F.Muell. | 0 | 0 | 0 | 3 | 7 | NSW883889 | DAR81578 |
| 16 | *Agonis flexuosa* (Willd.) Sweet (wild accession) ^g^ | 0 | 0 | 0 | 1 | 9 | NSW883854 | DAR81543 |
| 16 | *Agonis flexuosa* (Willd.) Sweet'Afterdark' ^g^ | 0 | 0 | 0 | 0 | 10 | NSW883853 | DAR81542 |
| 16 | *Kunzea ambigua* (Sm.) Druce hybrid ^g, n^ | 0 | 0 | 0 | 1 | 9 | NSW883917 | DAR81605 |
| 16 | *Kunzea ericoides* (A.Rich.) Joy Thomps. ^g^ | 0 | 0 | 0 | 0 | 10 | NSW883919 | DAR81607 |
| 16 | *Kunzea pomifera* F.Muell. ^g^ | 0 | 0 | 0 | 4 | 6 | NSW883920 | DAR81608 |
| 16 | *Leptospermum continentale* Joy Thomps.'Horizontalis' ^g^ | 0 | 0 | 0 | 2 | 8 | NSW883922 | DAR81610 |
| 16 | *Leptospermum* 'Lipstick' ^g, i^ | 0 | 0 | 0 | 2 | 8 | NSW883923 | DAR81611 |
| 16 | *Leptospermum* 'Rudolph' ^g^ | 0 | 0 | 0 | 0 | 10 | NSW883929 | DAR81616 |
| 16 | *Leptospermum* 'White Wave' ^g, k^ | 0 | 0 | 0 | 2 | 7 | NSW883930 | DAR81617 |
| 17 | *Chamelaucium uncinatum* Schauer ^g^ | 0 | 0 | 0 | 1 | 9 | NSW883870 | DAR81559 |
| 17 | *Darwinia citriodora* (Endl.) Benth. ^g, i^ | 0 | 0 | 0 | 2 | 8 | NSW883882 | DAR81571 |
| 17 | *Thryptomene calycina* (Lindl.) Stapf ^g^ | 0 | 0 | 0 | 4 | 6 | NSW883967 | DAR81653 |
| 17 | *Verticordia plumosa* (Desf.) Druce ^g^ | 0 | 0 | 0 | 1 | 9 | NSW883971 | DAR81657 |
|  | **Taxa with a range of symptoms** |  |  |  |  |  |  |  |
| 3 | *Xanthostemon chrysanthus* (F.Muell.) Benth. | 0 | 2 | 4 | 2 | 2 | NSW883972 | DAR81658 |
| 5 | *Osbornia octodonta* F.Muell. ^m^ | 1 | 1 | 3 | 3 | 4 | NSW883948 | DAR81634 |
| 6 | *Callistemon* 'Hannah Ray' ^g^, ^o^ | 1 | 0 | 0 | 3 | 4 | NSW883864 | DAR81553 |
| 6 | *Callistemon citrinus* (Curtis) Skeels'White Anzac' ^g, k^ | 2 | 0 | 7 | 0 | 0 | NSW883868 | DAR81557 |
| 6 | *Callistemon linearifolius* (Link) DC.^g^ | 0 | 0 | 1 | 3 | 6 | NSW883866 | DAR81555 |
| 6 | *Melaleuca ericifolia* Sm. ^k^ | 2 | 2 | 0 | 0 | 6 | NSW883940 | DAR81626 |
| 6 | *Melaleuca howeana* Cheel ^g, k, m^ | 1 | 0 | 0 | 2 | 1 | NSW883941 | DAR81627 |
| 6 | *Melaleuca quinquenervia* (Cav.) S.T.Blake ^g, k^ | 0 | 2 | 1 | 1 | 5 | NSW883943 | DAR81629 |
| 6 | *Regelia velutina* (Turcz.) C.A.Gardner | 2 | 0 | 0 | 2 | 6 | NSW883952 | DAR81639 |
| 7 | *Tristaniopsis laurina* (Sm.) Peter G.Wilson & J.T.Waterh. ^g^ | 5 | 2 | 0 | 0 | 3 | NSW883969 | DAR81655 |
| 8 | *Backhousia citriodora* F.Muell. ^g^ | 2 | 0 | 3 | 4 | 1 | NSW883860 | DAR81549 |
| 8 | *Backhousia myrtifolia* Hook. & Harv. ^k^ | 3 | 3 | 2 | 0 | 1 | NSW883861 | DAR81550 |
| 9 | *Metrosideros nervulosa* C.Moore & F.Muell. ^g, m^ | 1 | 0 | 0 | 0 | 4 | NSW883946 | DAR81632 |
| 11 | *Syzygium anisatum* (Vickery) Craven & Biffin ^g^ | 2 | 0 | 0 | 0 | 8 | NSW883955 | DAR81641 |
| 11 | *Syzygium australe* (J.C.Wendl. ex Link) B.Hyland'Captain Cook' ^p^ | 0 | 6 | 1 | 0 | 0 | NSW883956 | DAR81642 |
| 11 | *Syzygium fibrosum* (F.M.Bailey) T.G.Hartley & L.M.Perry | 0 | 8 | 2 | 0 | 0 | NSW883958 | DAR81644 |
| 11 | *Syzygium floribundum* F.Muell. ^g^ | 1 | 3 | 1 | 3 | 2 | NSW883959 | DAR81645 |
| 11 | *Syzygium francisii* (F.M.Bailey) L.A.S.Johnson ^g, i, o^ | 0 | 0 | 2 | 0 | 6 | NSW883960 | DAR81646 |
| 11 | *Syzygium fullagarii* (F.Muell.) Craven ^k, m^ | 1 | 0 | 0 | 2 | 1 | NSW883961 | DAR81647 |
| 11 | *Syzygium luehmannii* (F.Muell.) L.A.S.Johnson ^i^ | 3 | 1 | 4 | 0 | 2 | NSW883963 | DAR81649 |
| 11 | *Syzygium oleosum* (F.Muell.) B.Hyland ^o, q^ | 6 | 0 | 2 | 0 | 0 | NSW883964 | DAR81650 |
| 11 | *Syzygium smithii* (Poir.) Nied. rheophytic form ^g, k^ | 1 | 5 | 2 | 0 | 1 | NSW883965 | DAR81651 |
| 12 | *Decaspermum humile* (Sweet ex G.Don) A.J.Scott  ^g^ | 2 | 0 | 6 | 2 | 0 | NSW883883 | DAR81572 |
| 12 | *Pimenta dioica* (L.) Merr. ^h, k^ | 0 | 0 | 5 | 1 | 3 | NSW883949 | DAR81635 |
| 13 | *Allosyncarpia ternata* S.T.Blake ^k, p^ | 0 | 5 | 1 | 0 | 0 | NSW883855 | DAR81544 |
| 13 | *Angophora costata* (Gaertn.) Britten ^g^ | 1 | 3 | 3 | 2 | 1 | NSW883856 | DAR81545 |
| 13 | *Angophora floribunda* (Sm.) Sweet ^g^ | 0 | 3 | 4 | 2 | 1 | NSW883857 | DAR81546 |
| 13 | *Corymbia citriodora* (Hook.) K.D.Hill & L.A.S.Johnson (accession no 1) | 5 | 3 | 1 | 0 | 1 | NSW883872 | DAR81561 |
| 13 | *Corymbia citriodora* (Hook.) K.D.Hill & L.A.S.Johnson (accession no 2) ^r, s^ | 4 | 0 | 2 | 2 | 0 | NSW883880 | DAR81569 |
| 13 | *Corymbia ficifolia* (F.Muell.) K.D.Hill & L.A.S.Johnson | 5 | 1 | 2 | 2 | 0 | NSW883873 | DAR81562 |
| 13 | *Corymbia gummifera* (Gaertn.) K.D.Hill & L.A.S.Johnson ^o^ | 4 | 1 | 2 | 1 | 0 | NSW883874 | DAR81563 |
| 13 | *Corymbia henryi* (S.T.Blake) K.D.Hill & L.A.S.Johnson ^g^ | 0 | 1 | 4 | 0 | 5 | NSW883875 | DAR81564 |
| 13 | *Corymbia intermedia* (R.T.Baker) K.D.Hill & L.A.S.Johnson ^g^ | 3 | 3 | 3 | 0 | 1 | NSW883876 | DAR81565 |
| 13 | *Corymbia maculata* (Hook.) K.D.Hill & L.A.S.Johnson | 4 | 3 | 2 | 1 | 0 | NSW883877 | DAR81566 |
| 13 | *Corymbia tessellaris* (F.Muell.) K.D.Hill & L.A.S.Johnson ^g^ | 3 | 6 | 1 | 0 | 0 | NSW883878 | DAR81567 |
| 13 | *Corymbia torelliana* (F.Muell.) K.D.Hill & L.A.S.Johnson | 2 | 1 | 7 | 0 | 0 | NSW883879 | DAR81568 |
| 13 | *Corymbia variegata* × *torelliana* | 0 | 6 | 2 | 1 | 1 | NSW883881 | DAR81570 |
| 13 | *Eucalyptus agglomerata* Maiden ^g^ | 0 | 2 | 3 | 1 | 4 | NSW883884 | DAR81573 |
| 13 | *Eucalyptus argophloia* Blakely | 7 | 0 | 1 | 2 | 0 | NSW883886 | DAR81575 |
| 13 | *Eucalyptus campanulata* R.T.Baker & H.G.Sm. ^t^ | 1 | 2 | 6 | 1 | 0 | NSW883885 | DAR81574 |
| 13 | *Eucalyptus cladocalyx* F.Muell. ^k^ | 4 | 3 | 2 | 0 | 0 | NSW883888 | DAR81577 |
| 13 | *Eucalyptus diversicolor* F.Muell. | 0 | 1 | 9 | 0 | 0 | NSW883890 | DAR81579 |
| 13 | *Eucalyptus dunnii* Maiden | 3 | 0 | 6 | 1 | 0 | NSW883892 | DAR81581 |
| 13 | *Eucalyptus globulus* Labill. subsp. *bicostata* (Maiden, Blakely & Simmonds) J.B.Kirkp. ^i^ | 2 | 2 | 2 | 0 | 4 | NSW883893 | DAR81582 |
| 13 | *Eucalyptus globulus* Labill. subsp. *globulus* ^k, i, r^ | 3 | 1 | 1 | 0 | 2 | NSW883894 | DAR81583 |
| 13 | *Eucalyptus gomphocephala* A.Cunn. ex DC. | 5 | 1 | 1 | 2 | 1 | NSW883895 | DAR81584 |
| 13 | *Eucalyptus grandis* W.Hill ^k^ | 1 | 2 | 5 | 2 | 0 | NSW883896 | DAR81585 |
| 13 | *Eucalyptus haemastoma* Sm. | 3 | 0 | 4 | 0 | 3 | NSW883898 | DAR81586 |
| 13 | *Eucalyptus laevopinea* R.T.Baker ^k, r^ | 2 | 1 | 2 | 0 | 2 | NSW883899 | DAR81587 |
| 13 | *Eucalyptus longirostrata* (Blakely) L.A.S.Johnson & K.D.Hill ^u^ | 0 | 5 | 5 | 0 | 0 | NSW883900 | DAR81588 |
| 13 | *Eucalyptus marginata* D.Don ex Sm. subsp. *marginata* ^k^ | 1 | 3 | 3 | 1 | 1 | NSW883901 | DAR81589 |
| 13 | *Eucalyptus nitens* (H.Deane & Maiden) Maiden ^g, i^ | 1 | 1 | 2 | 0 | 6 | NSW883903 | DAR81590 |
| 13 | *Eucalyptus obliqua* L'Her. ^k, m^ | 0 | 3 | 0 | 0 | 1 | — | DAR81591 |
| 13 | *Eucalyptus occidentalis* Endl. ^g^ | 2 | 2 | 3 | 1 | 2 | NSW883904 | DAR81592 |
| 13 | *Eucalyptus olida* L.A.S.Johnson & K.D.Hill | 7 | 0 | 1 | 0 | 2 | NSW883905 | DAR81593 |
| 13 | *Eucalyptus pellita* F.Muell. ^p^ | 2 | 4 | 0 | 1 | 0 | NSW883906 | DAR81594 |
| 13 | *Eucalyptus pilularis* Sm. | 0 | 5 | 4 | 0 | 1 | NSW883907 | DAR81595 |
| 13 | *Eucalyptus populnea* F.Muell. | 1 | 4 | 1 | 2 | 2 | NSW883908 | DAR81596 |
| 13 | *Eucalyptus punctata* DC ^v^ | 1 | 3 | 4 | 0 | 2 | NSW883887 | DAR81576 |
| 13 | *Eucalyptus regnans* F.Muell. | 0 | 1 | 6 | 0 | 3 | NSW883909 | DAR81597 |
| 13 | *Eucalyptus resinifera* subsp. *hemilampra* (F.Muell.) L.A.S.Johnson & K.D.Hill | 4 | 0 | 6 | 0 | 0 | NSW883910 | DAR81598 |
| 13 | *Eucalyptus saligna* Sm. | 3 | 3 | 4 | 0 | 0 | NSW883911 | DAR81599 |
| 13 | *Eucalyptus siderophloia* Benth. ^w^ | 5 | 3 | 2 | 0 | 0 | NSW883891 | DAR81580 |
| 13 | *Eucalyptus smithii* R.T.Baker | 1 | 1 | 7 | 1 | 0 | NSW883912 | DAR81600 |
| 13 | *Eucalyptus tereticornis* Sm. ^k, x^ | 6 | 0 | 3 | 0 | 0 | NSW883913 | DAR81601 |
| 13 | *Eucalyptus tindaliae* Blakely in J.H.Maiden ^k^ | 2 | 0 | 5 | 1 | 1 | NSW883914 | DAR81602 |
| 13 | *Eucalyptus wandoo* Blakely subsp. wandoo | 1 | 1 | 1 | 1 | 6 | NSW883915 | DAR81603 |
| 14 | *Syncarpia glomulifera* (Sm.) Nied. ^g^ | 2 | 2 | 5 | 0 | 1 | NSW883954 | DAR81640 |
| 15 | *Lindsayomyrtus racemoides* (Greves) Craven ^g, i, m^ | 0 | 0 | 2 | 0 | 3 | NSW883936 | DAR81623 |
| 16 | *Asteromyrtus magnifica* (Specht) Craven ^k^ | 5 | 0 | 0 | 3 | 1 | NSW883858 | DAR81547 |
| 16 | *Kunzea baxteri* (Klotzsch) Schauer ^g^ | 0 | 0 | 1 | 0 | 9 | NSW883918 | DAR81606 |
| 16 | *Leptospermum* 'Day Dream' ^g^ | 4 | 0 | 0 | 3 | 3 | NSW883921 | DAR81609 |
| 16 | *Leptospermum* 'Love Affair' ^g^ | 1 | 0 | 0 | 0 | 9 | NSW883924 | DAR81612 |
| 16 | *Leptospermum* 'Mesmer Eyes' ^g, r^ | 0 | 0 | 2 | 1 | 5 | NSW883925 | DAR81613 |
| 16 | *Leptospermum* 'Pink Cascade' | 9 | 0 | 1 | 0 | 0 | NSW883926 | — |
| 16 | *Leptospermum* 'Rhiannon' ^g^ | 1 | 0 | 0 | 2 | 7 | NSW883927 | DAR81614 |
| 16 | *Leptospermum* 'Riot' ^g, o^ | 2 | 0 | 2 | 2 | 2 | NSW883928 | DAR81615 |
| 16 | *Leptospermum laevigatum* (Gaertn.) F.Muell. ^i, k^ | 2 | 0 | 0 | 3 | 4 | NSW883931 | DAR81618 |
| 16 | *Leptospermum morrisonii* Joy Thomps.'Burgundy' ^g, o^ | 2 | 0 | 0 | 2 | 4 | NSW883932 | DAR81619 |
| 16 | *Leptospermum polygalifolium* Salisb. ^g, o^ | 1 | 0 | 0 | 3 | 4 | NSW883933 | DAR81620 |
| 16 | *Leptospermum polygalifolium* × *scoparium* ^g^ | 5 | 0 | 0 | 1 | 4 | NSW883934 | DAR81621 |
| 16 | *Leptospermum trinervium* (Sm.) Joy Thomps. ^g, k, r^ | 2 | 0 | 0 | 0 | 5 | NSW883935 | DAR81622 |
| 17 | *Verticordia chrysantha* Endl. ^g^ | 4 | 0 | 0 | 4 | 2 | NSW883970 | DAR81656 |

^a^ Tribe number based on Wilson et al. [29].

^b^ It is the authors understanding that where varieties are listed for a taxon they are clonally propagated with the exception of *P. guajava* 'Hawaiian', *P. guajava* 'Indian' and *C. calophylla* ‘Rosea’. In contrast all taxa labelled at the species level are believed to be of seedling origin.

^c^ Five replicates of each taxon were used in each of the two trials performed unless indicated otherwise. Pooled results from the trials are presented.

^d^ Voucher of a representative plant used in tests lodged at the National Herbarium of NSW, Royal Botanic Gardens Sydney.

^e^ Diseased specimens lodged at the Plant Pathology Herbarium (HERB-DAR) of the NSW Department of Primary Industries.

^f^ Number of replicates in each disease category across all 23 trials performed. Six replicates of *S. jambos* per trial were used.

^g^ Uredinia present on the stem of at least one replicate.

^h^ Exotic species.

^i^ Teliospores present on one or more replicates.

^j^ No voucher specimen lodged.

^k^ Four replicates were used in one of the trials for this taxon.

^l^ Originates from *Metrosideros excelsa* ‘Pink Lady’.

^m^ Only one trial performed.

^n^ Hybrid, but other parent unknown.

^o^ Four replicates were used in both trials for this taxon.

^p^ Two replicates were used in one of the trials for this taxon.

^q^ Purchased as *Syzygium paniculatum* but identified as *S. oleosum*.

^r^ Three replicates were used in one of the trials for this taxon.

^s^ Supplied as *Corymbia variegata* (F.Muell.) K.D.Hill & L.A.S.Johnson, which is considered a synonym of *C. citriodora* in APC.

^t^ Supplied as *Eucalyptus andrewsii* Maiden, but identified as the related species *E. campanulata*, which is recognized by some as *Eucalyptus andrewsii* subsp. *campanulata* (R.T.Baker & H.G.Sm.) L.A.S.Johnson & Blaxell.

^u^ Supplied as *E. longirostrata*, but identification uncertain. It is likely to be a related species of Grey Gum.

^v^ Supplied as *Eucalyptus biturbinata* L.A.S.Johnson & K.D.Hill, which is considered a synonym of *E. punctata* in APC.

^w^ Originally misidentified as *Eucalyptus drepanophylla* F.Muell. ex Benth.

^x^ Supplied as *E. tereticornis*, but identification uncertain.
