# Supplementary material for: Investigating the Host-Range of the Rust Fungus Puccinia psidii sensu lato across Tribes of the Family Myrtaceae Present in Australia
Source: PLoS One. 2012 Apr 16;7(4):e35434. doi: 10.1371/journal.pone.0035434 (PMC3327671; doi:10.1371/journal.pone.0035434)
Supplement: Figure S1 — Photographs of the most developed uredinia produced on the various taxa. Photographs of the most developed uredinia produced on the various taxa at three weeks after inoculation with Puccinia psidii s.l. (ex Australia, DAR81284). (PDF) [file pone.0035434.s002.pdf]

Figure S1. Photographs of the most developed uredinia produced on the various taxa.

Photographs of the most developed uredinia produced on the various taxa at three weeks after inoculation with *Puccinia psidii* s.l. (ex Australia, DAR81284).

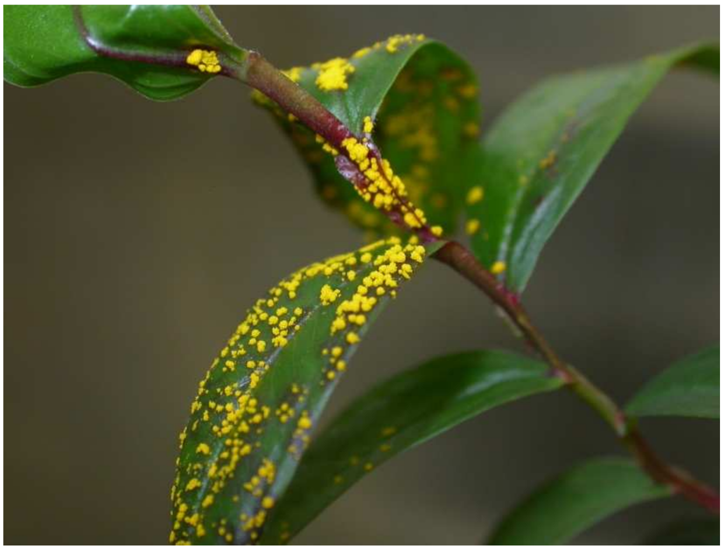

Agonis flexuosa 'Afterdark'

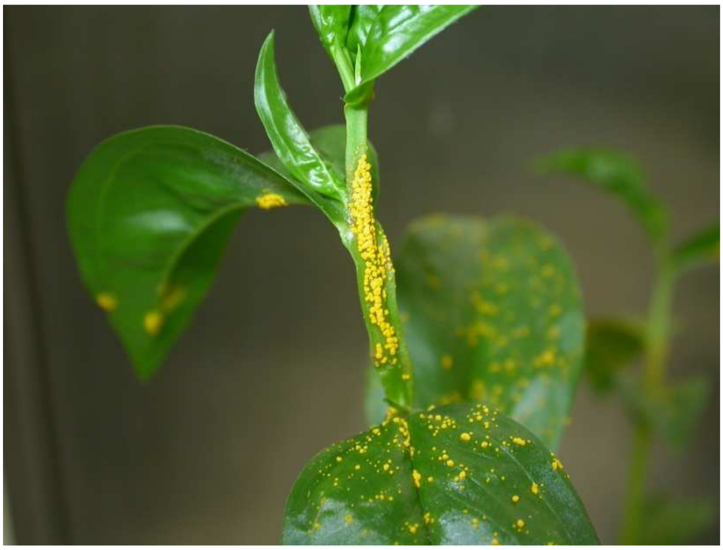

Agonis flexuosa (wild accession)

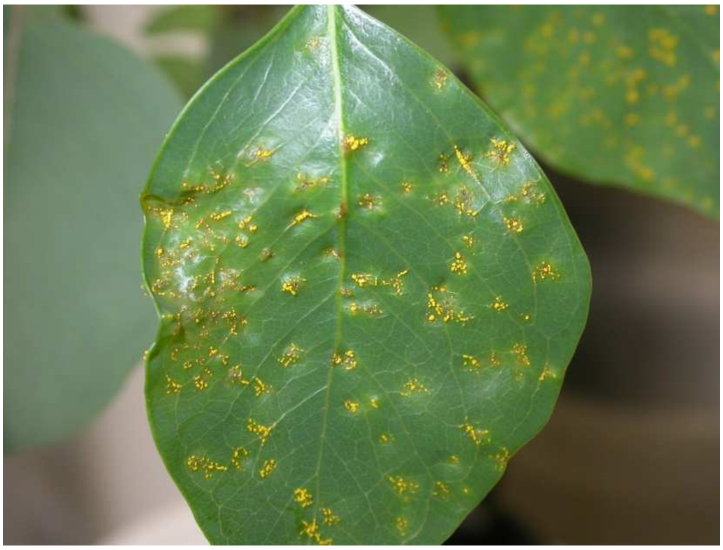

Allosyncarpia ternata

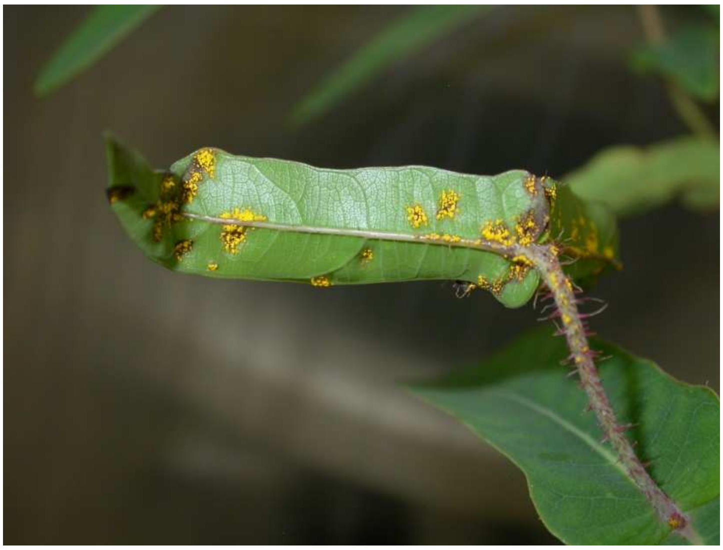

Angophora costata

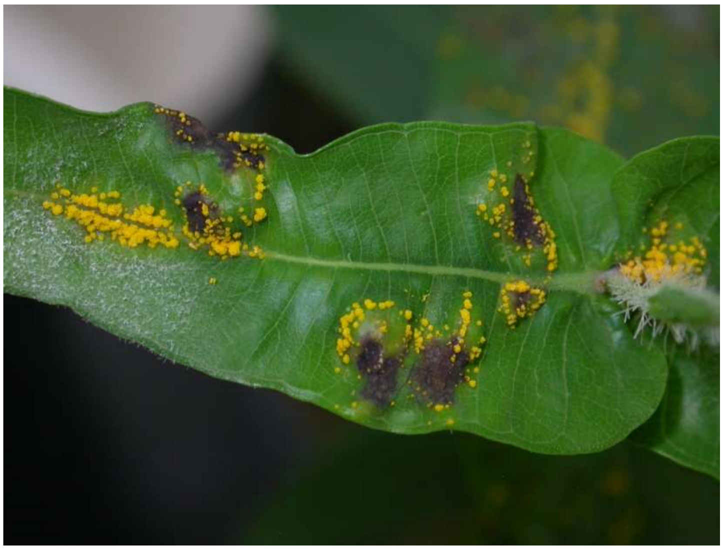

Angophora floribunda

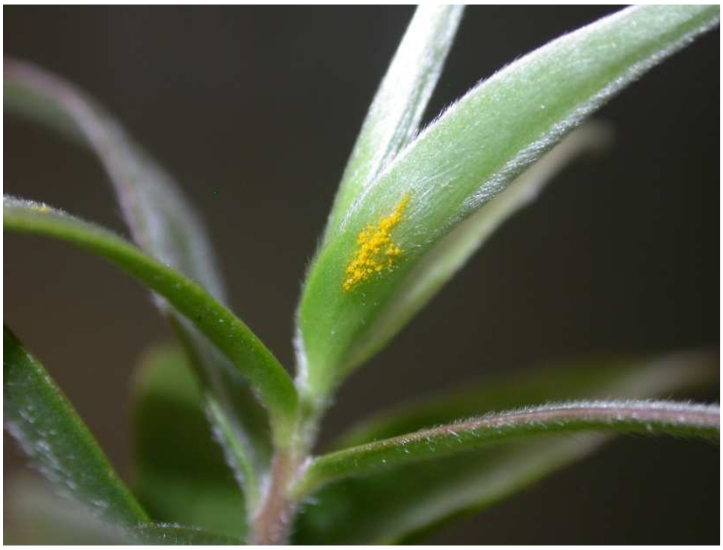

Asteromyrtus magnifica

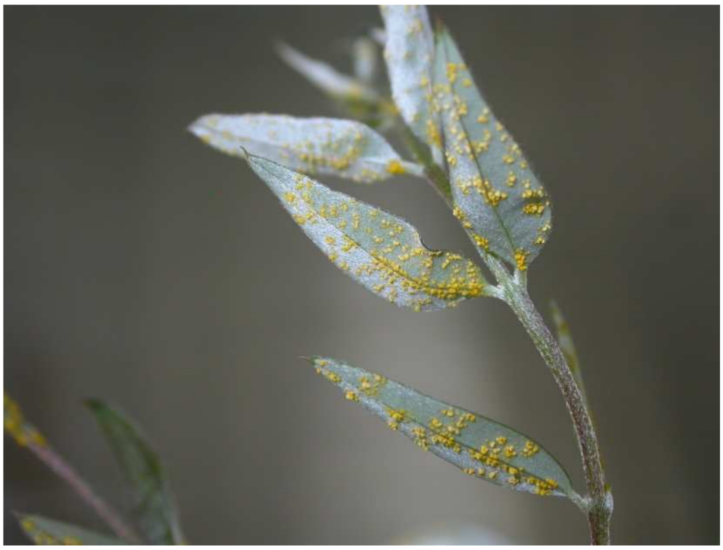

Austromyrtus dulcis

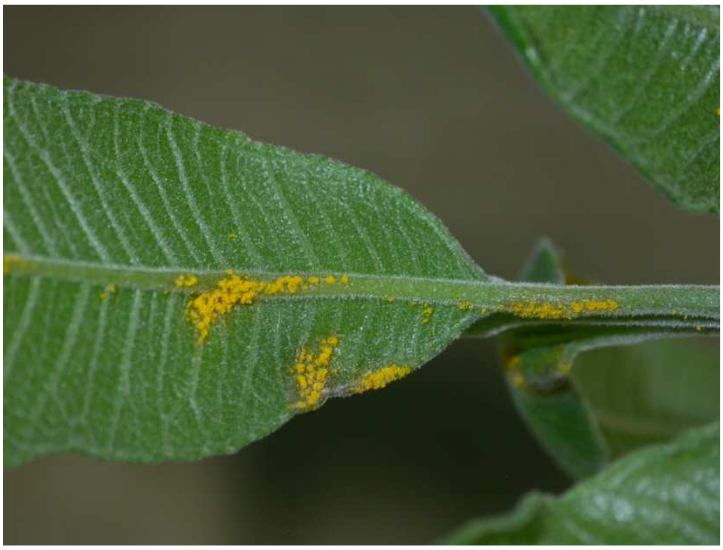

Backhousia citriodora

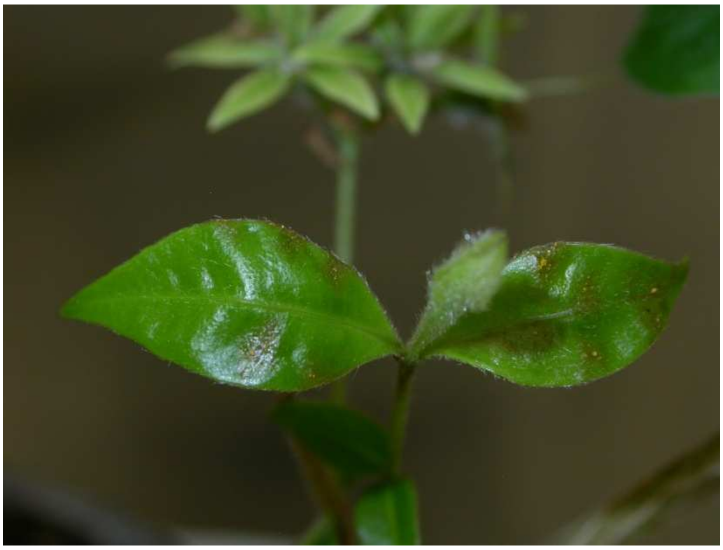

Backhousia myrtifolia

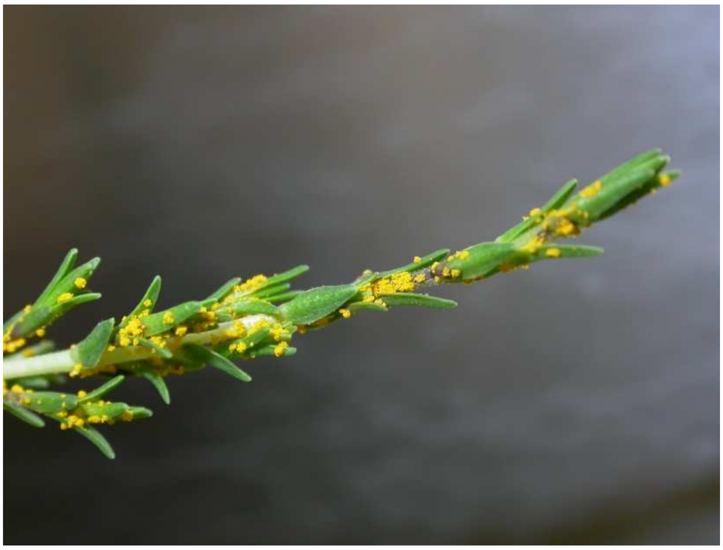

Beaufortia schaueri

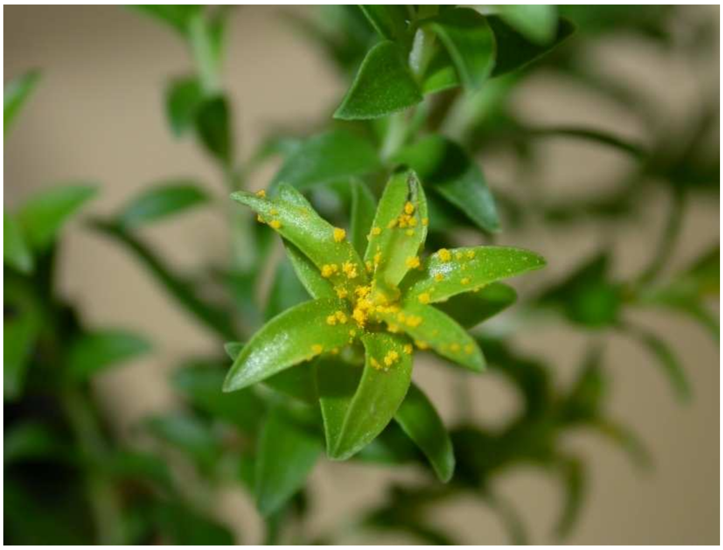

Beaufortia sparsa

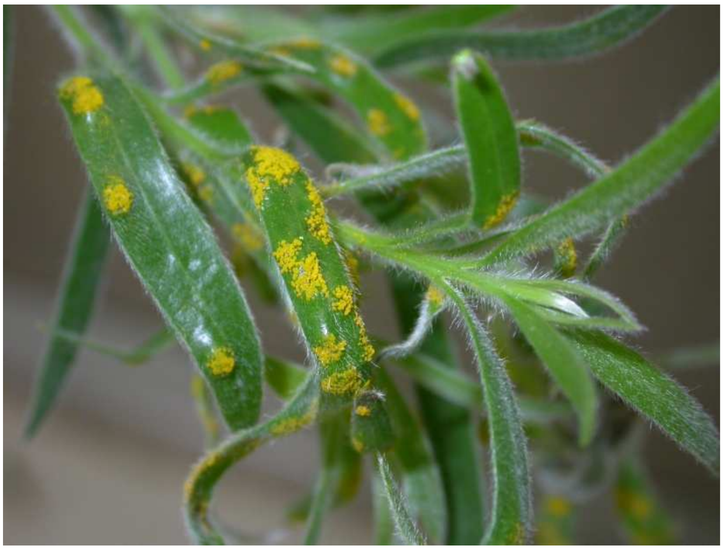

Callistemon 'Hannah Ray'

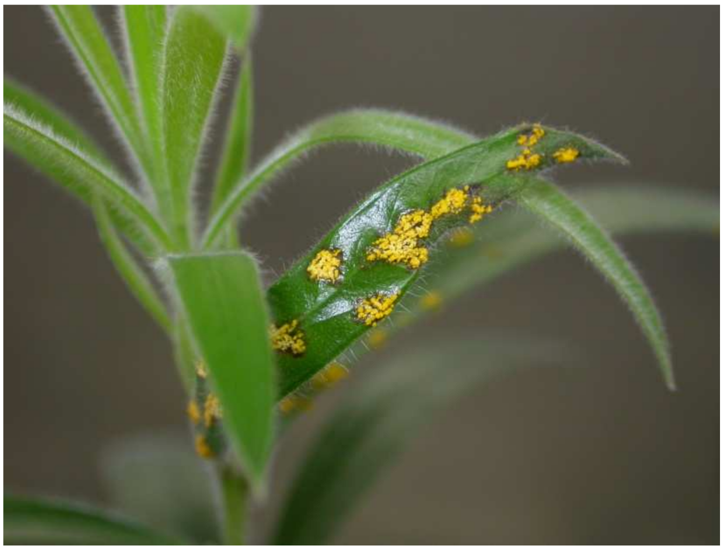
Callistemon 'Kings Park Special'

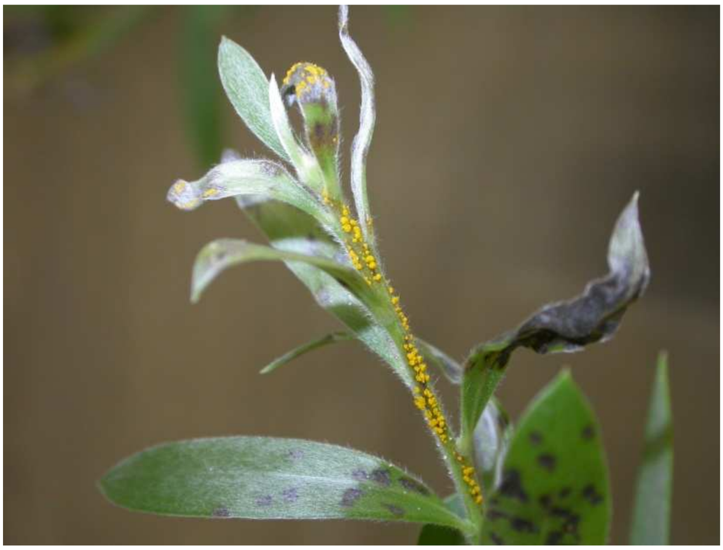
Callistemon citrinus 'White Anzac'

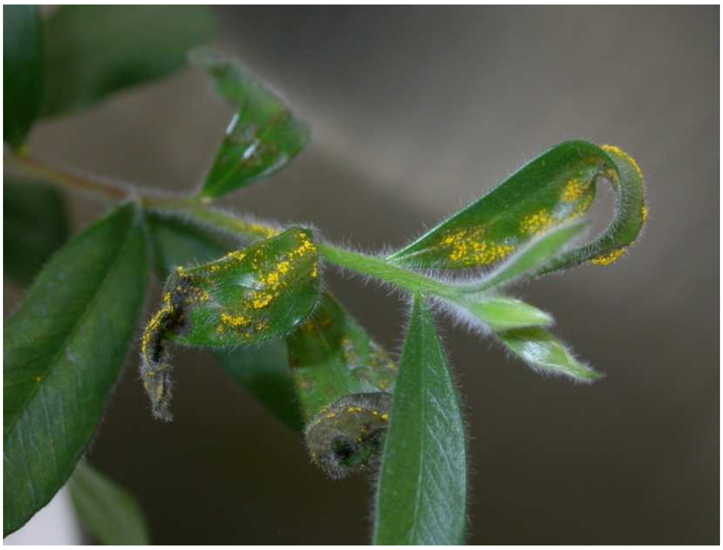
Callistemon linearifolius

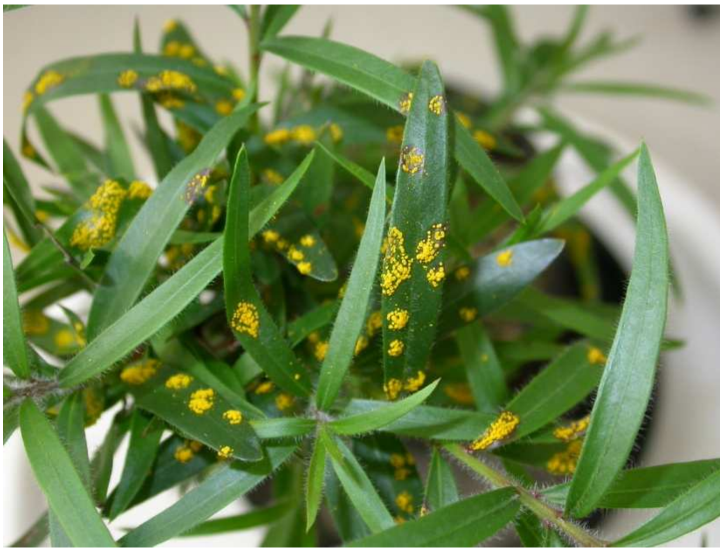
Callistemon viminalis

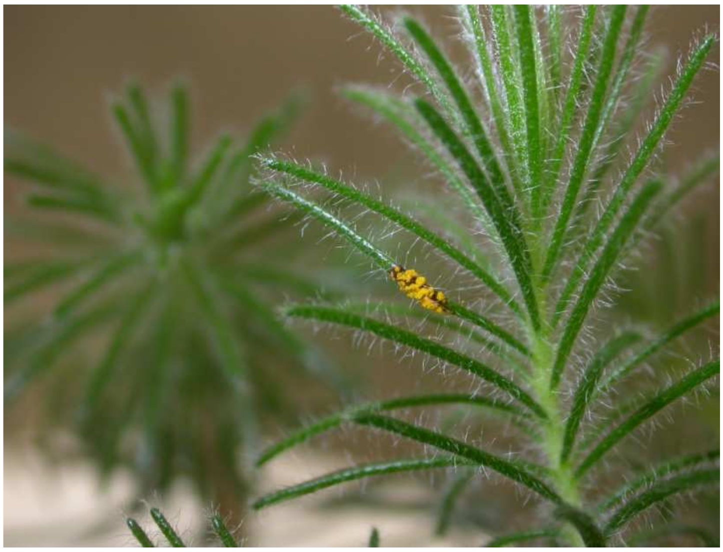
Calothamnus quadrifidus

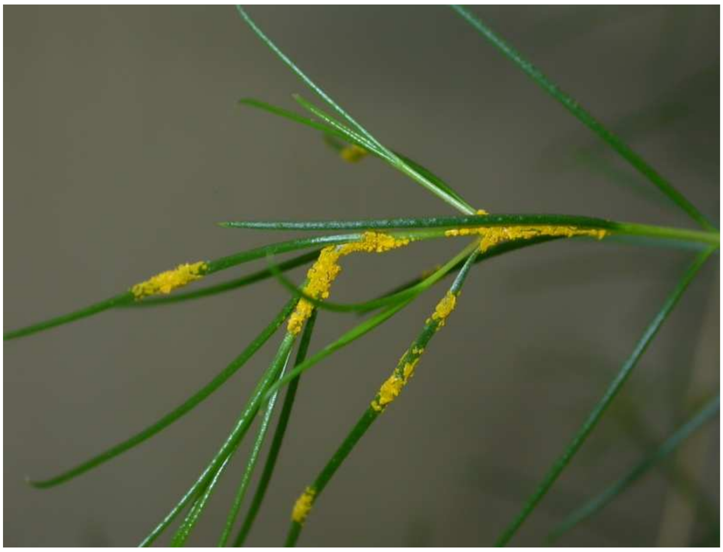
Chamelaucium uncinatum

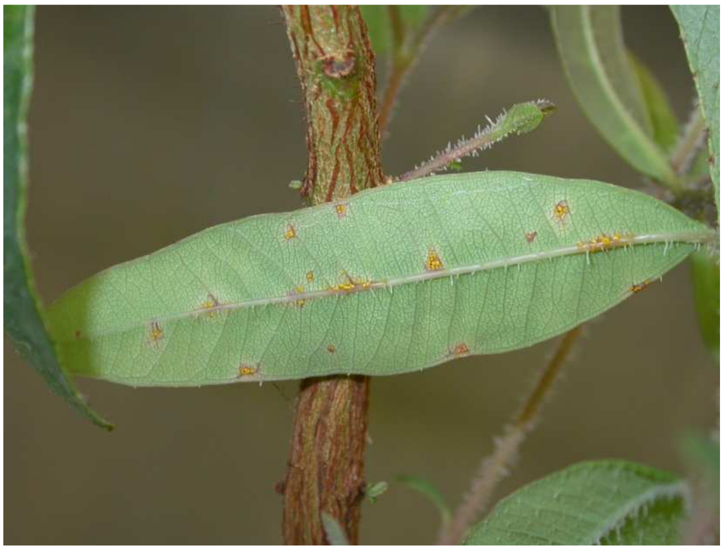

Corymbia citriodora (accession no 1)

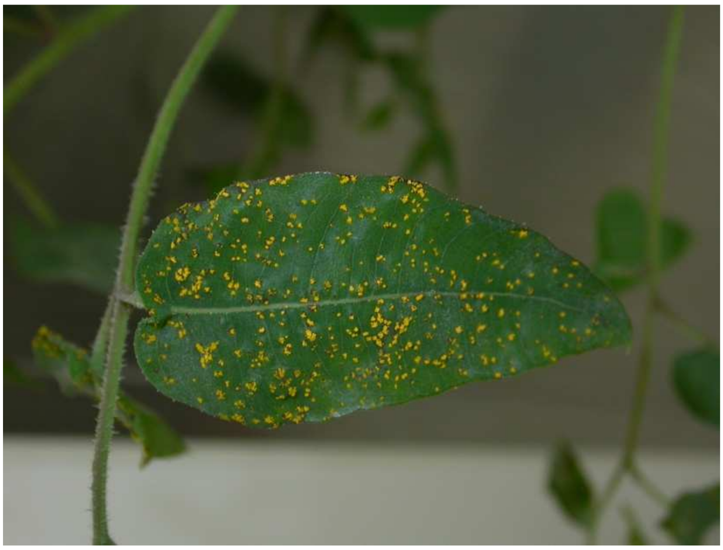

Corymbia citriodora (accession no 2)

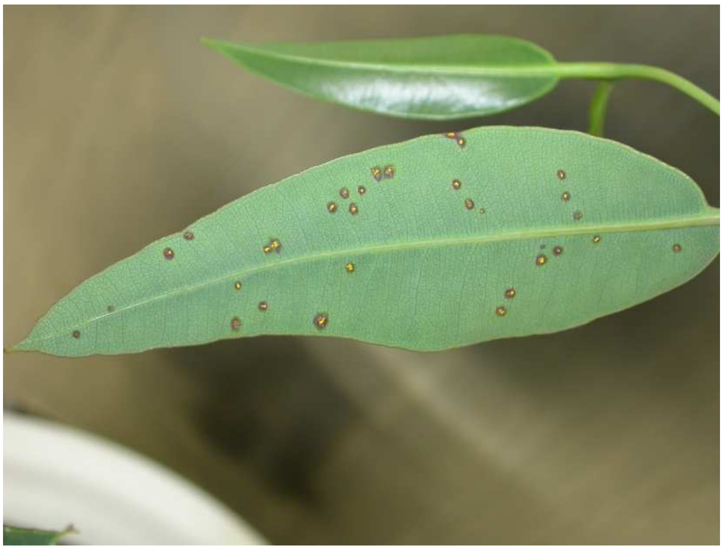

Corymbia ficifolia

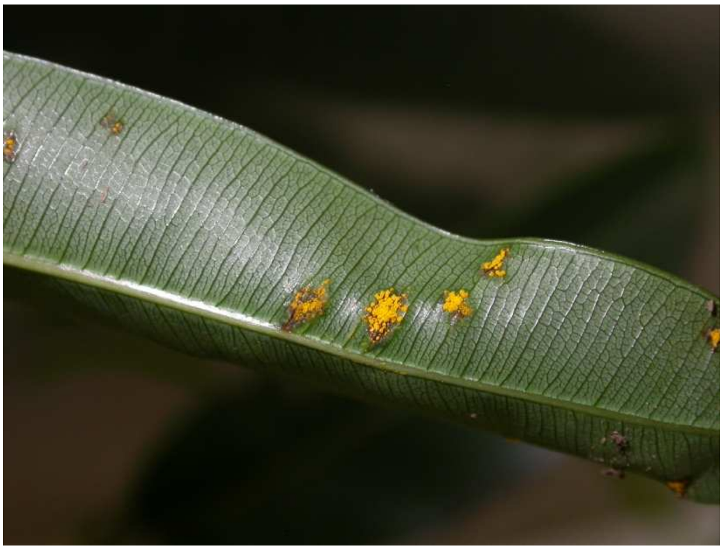

Corymbia gummifera

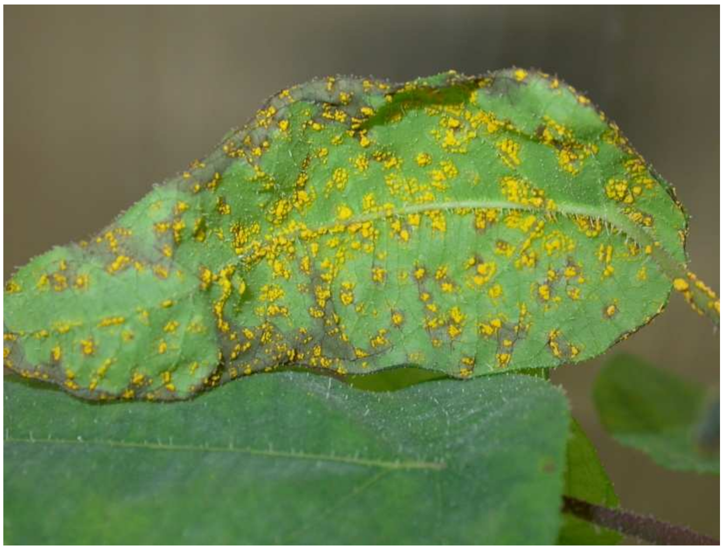

Corymbia henryi

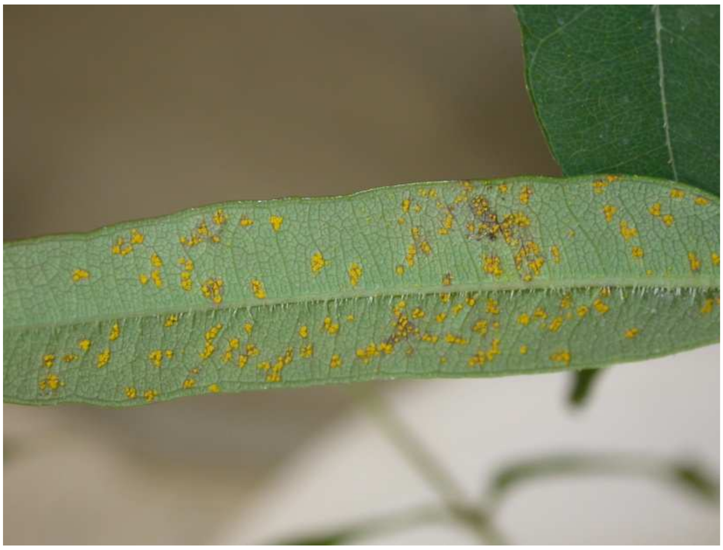

Corymbia intermedia

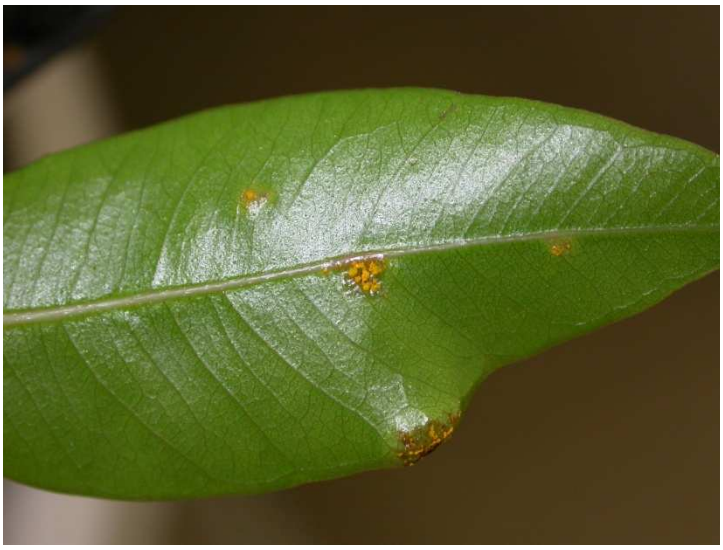

Corymbia maculata

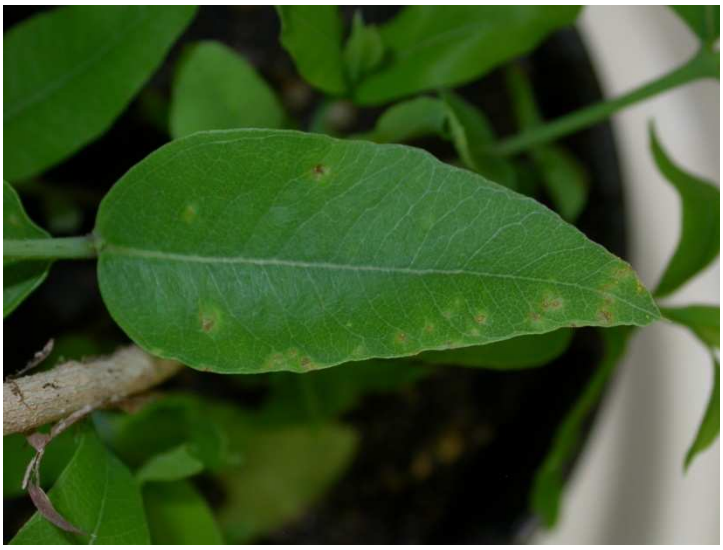

Corymbia tessellaris

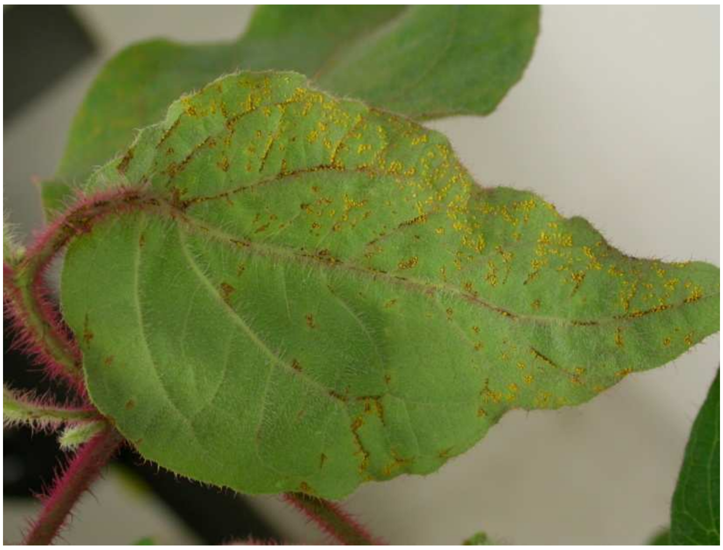

Corymbia torelliana

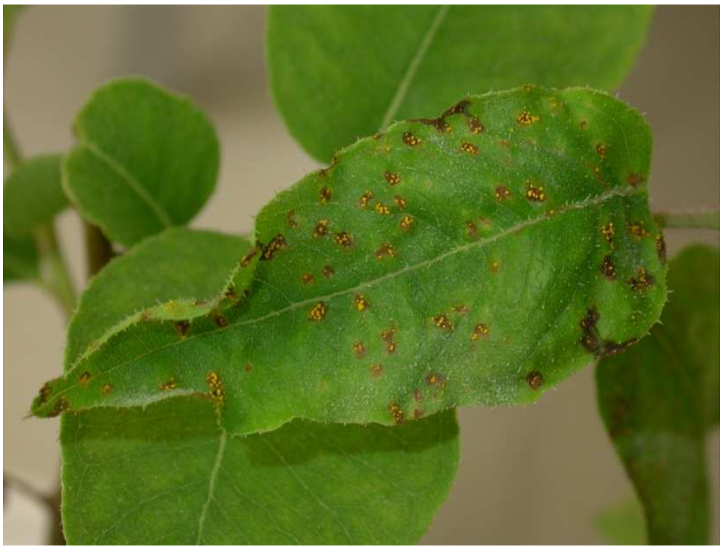

Corymbia variegata × torelliana

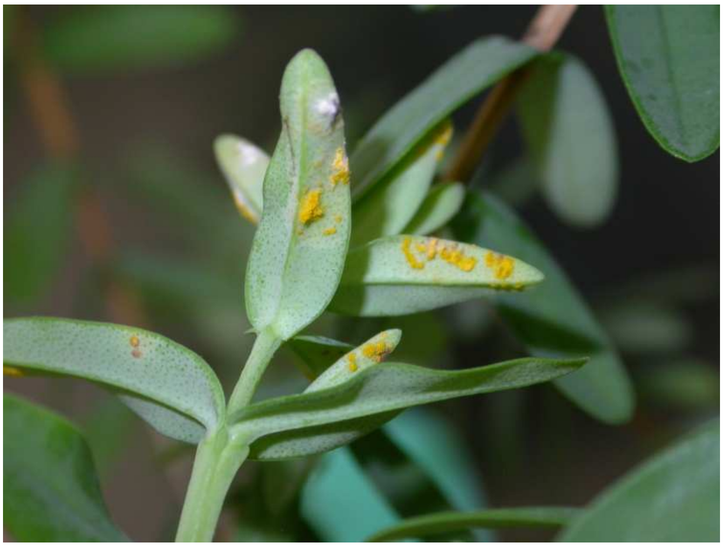

Darwinia citriodora

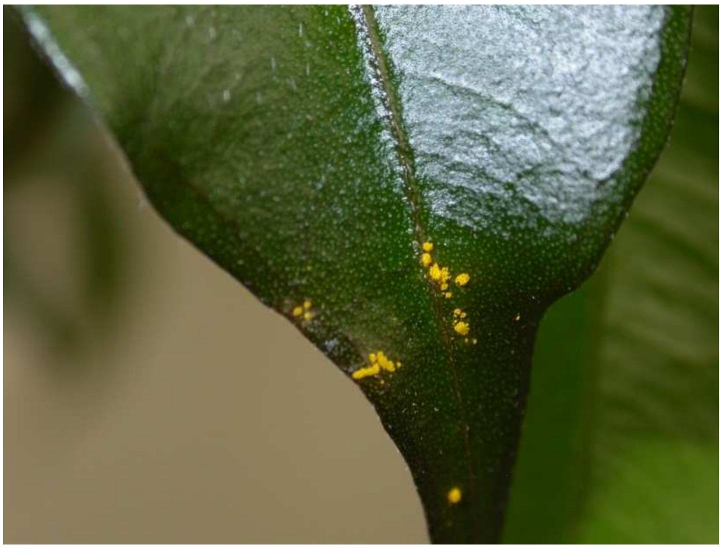

Decaspermum humile

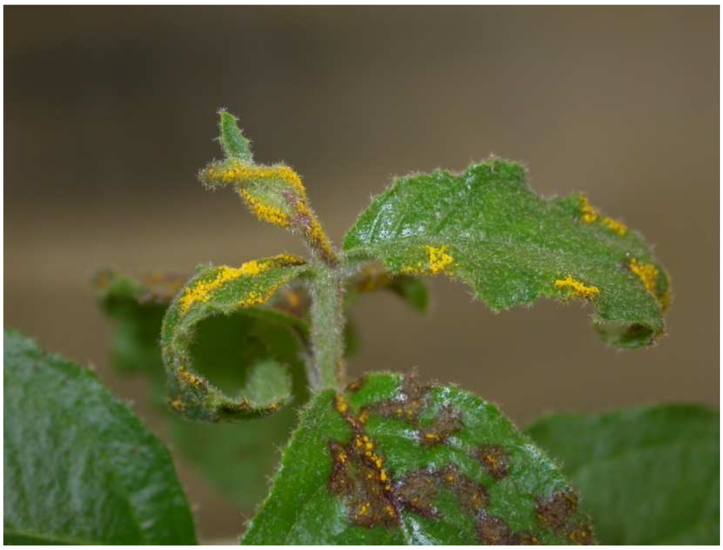

Eucalyptus agglomerata

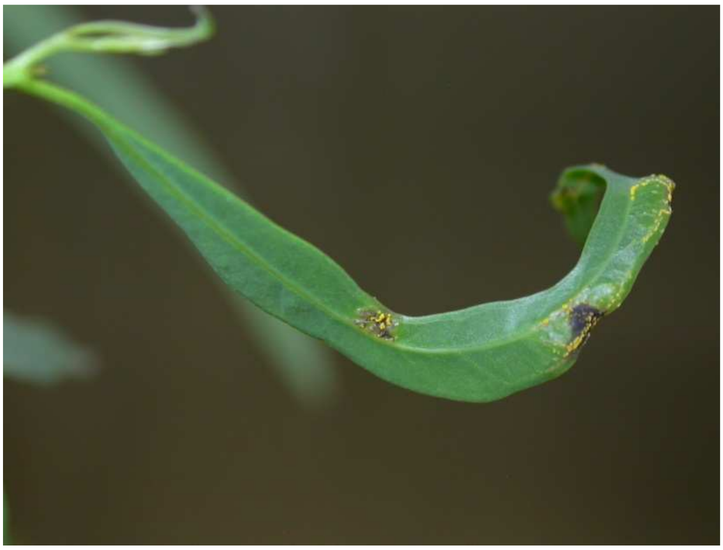

Eucalyptus argophloia

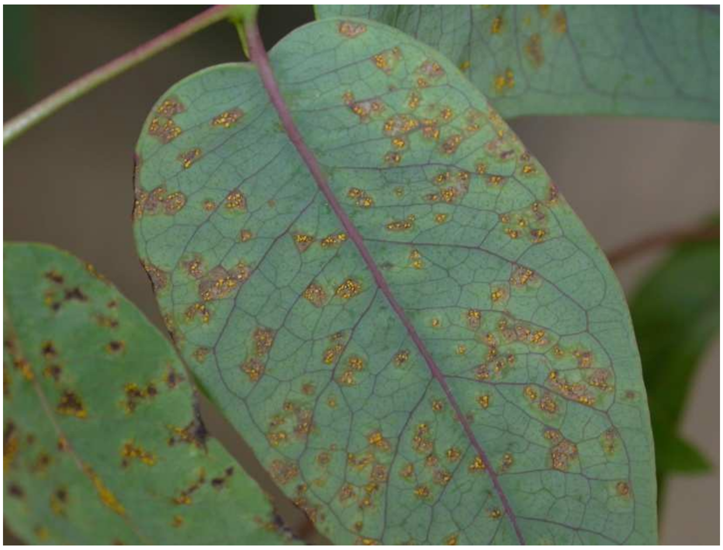

Eucalyptus campanulata

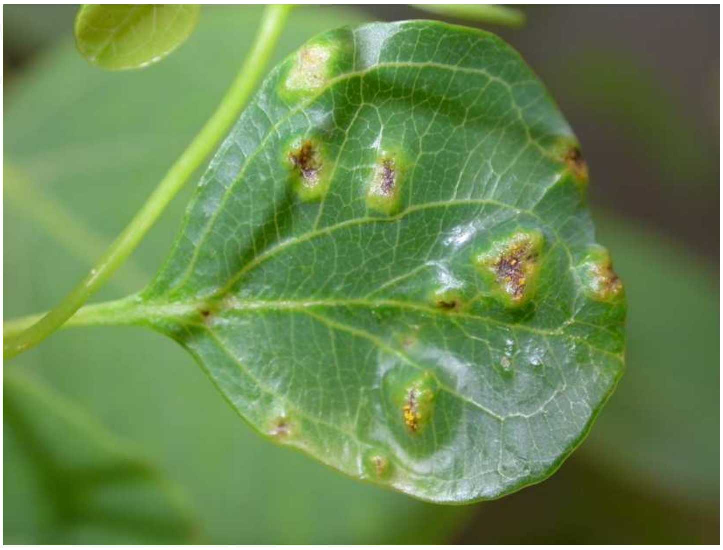

Eucalyptus cladocalyx

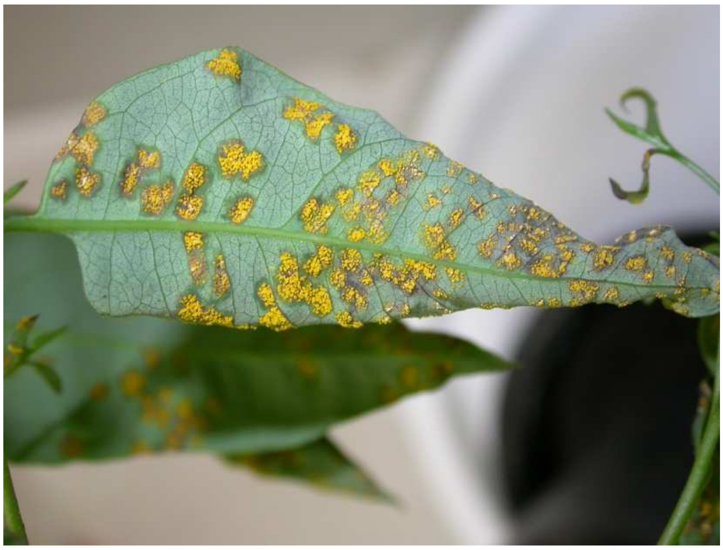

Eucalyptus cloeziana

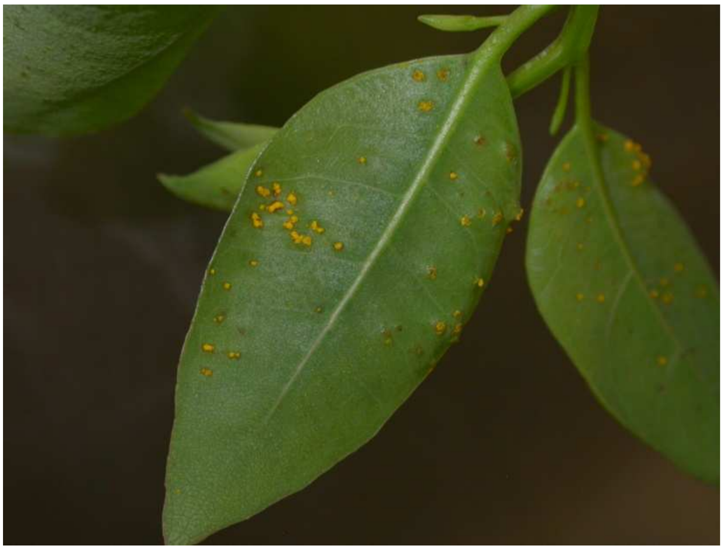

Eucalyptus diversicolor

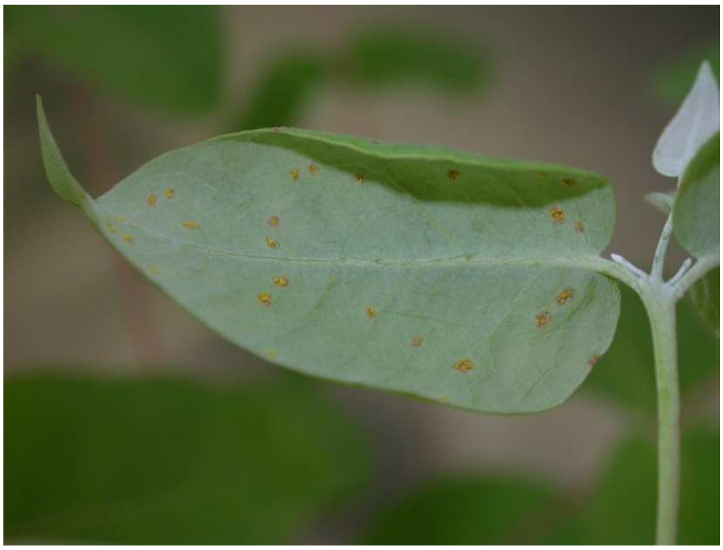

Eucalyptus dunnii

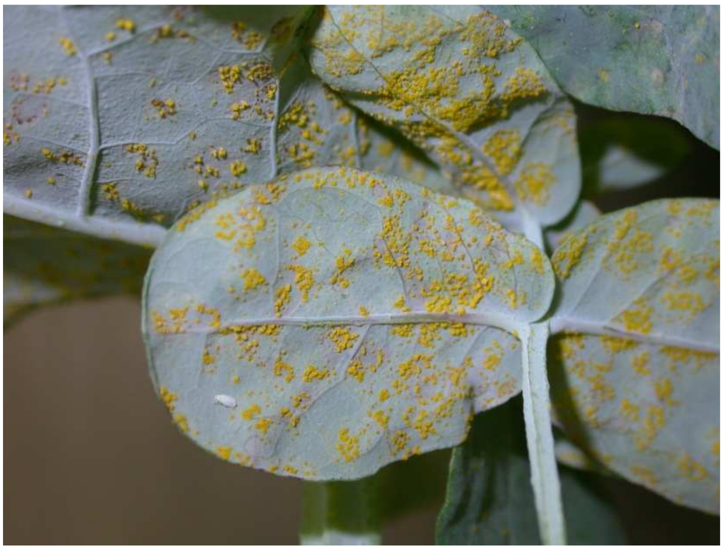

Eucalyptus globulus subsp. *bicostata*

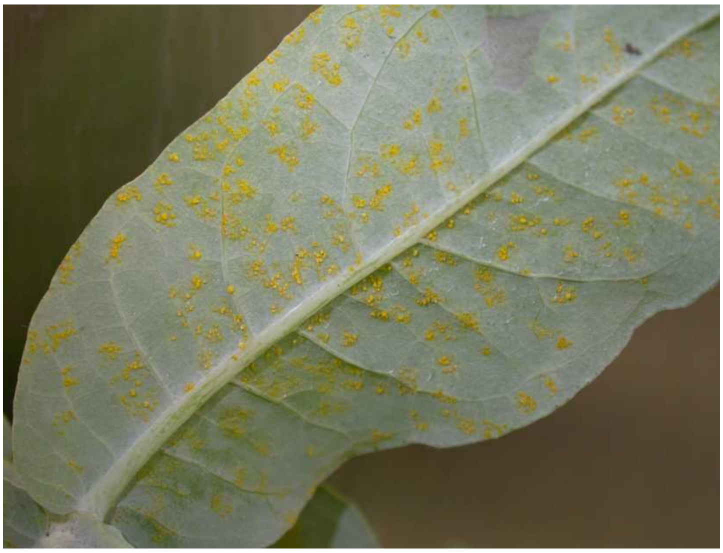

Eucalyptus globulus subsp. *globulus*

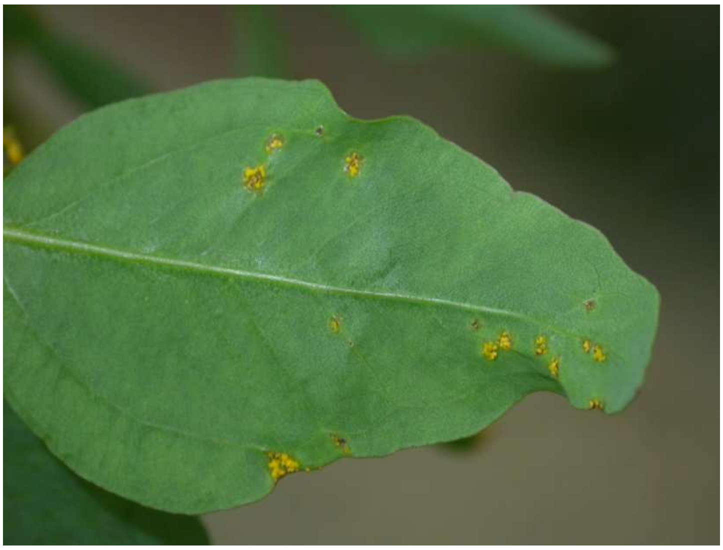

Eucalyptus gomphocephala

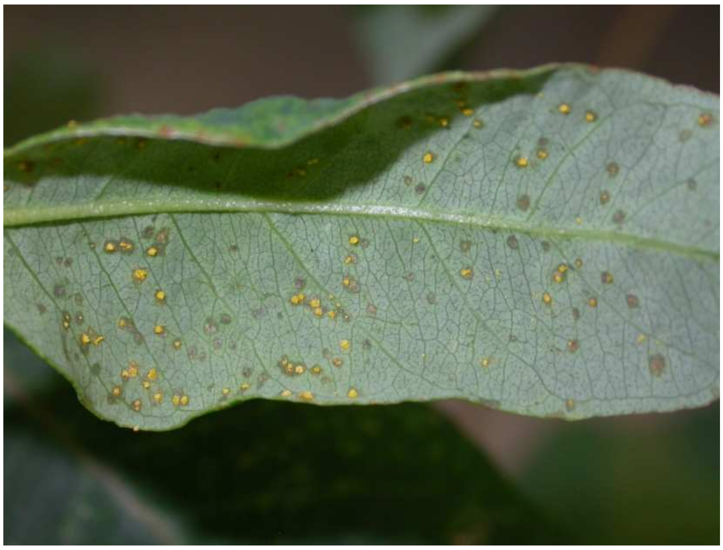

Eucalyptus grandis

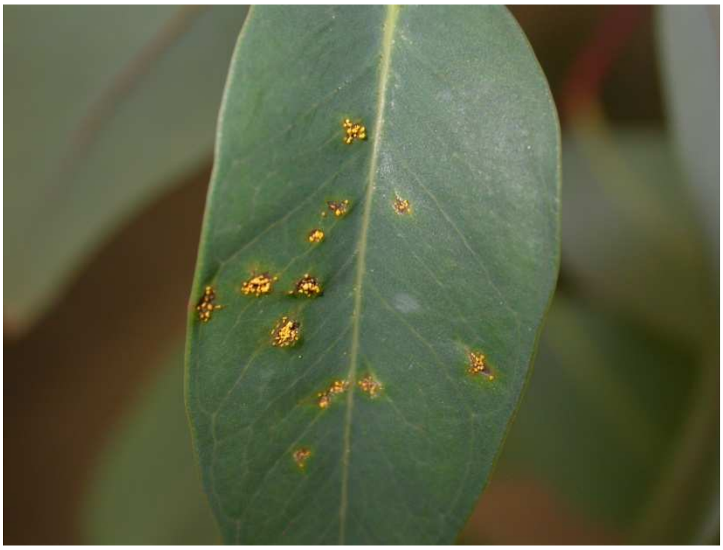

Eucalyptus haemastoma

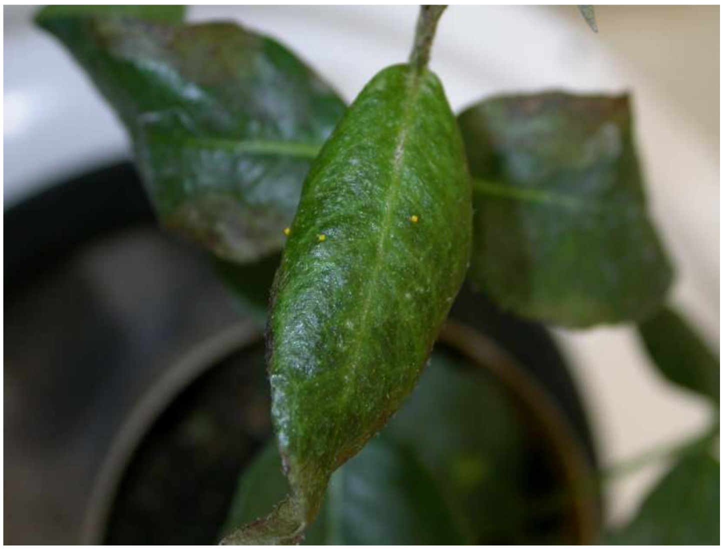

Eucalyptus laevopinea

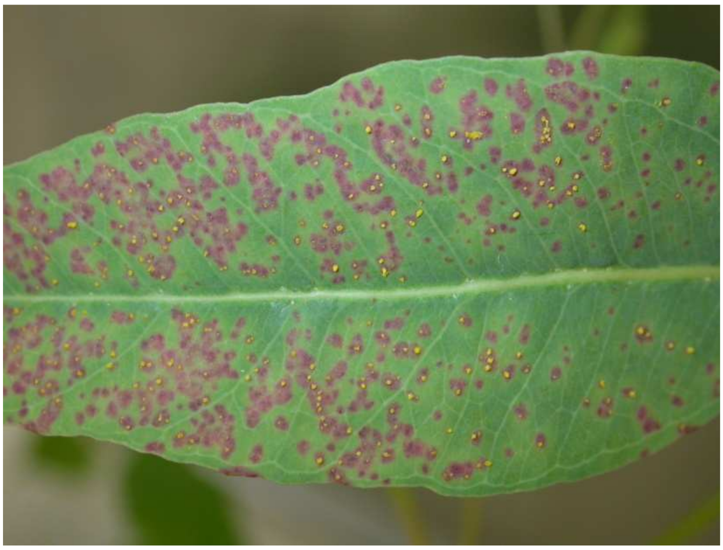

Eucalyptus longirostrata

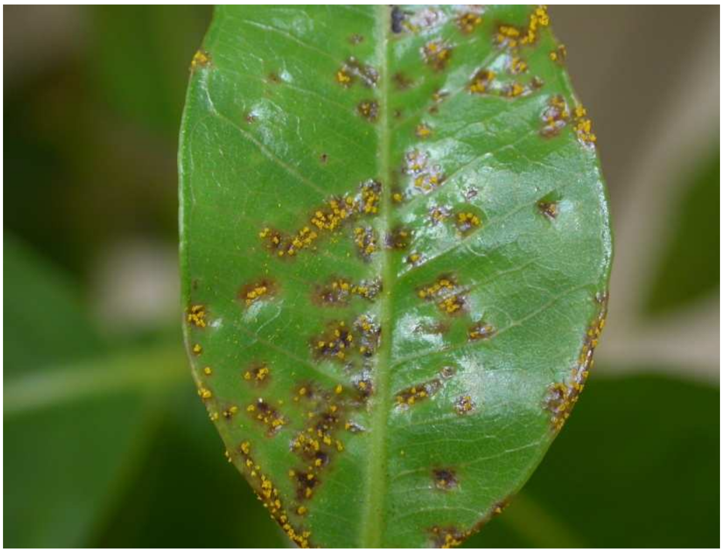

Eucalyptus marginata subsp. *marginata*

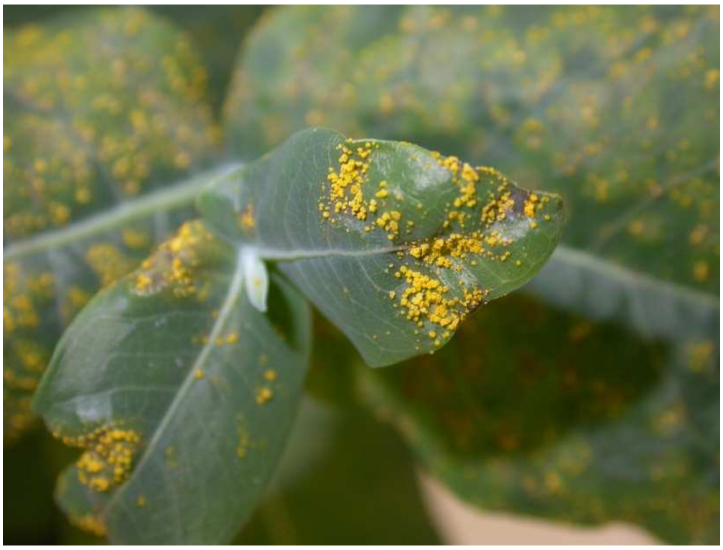

Eucalyptus nitens

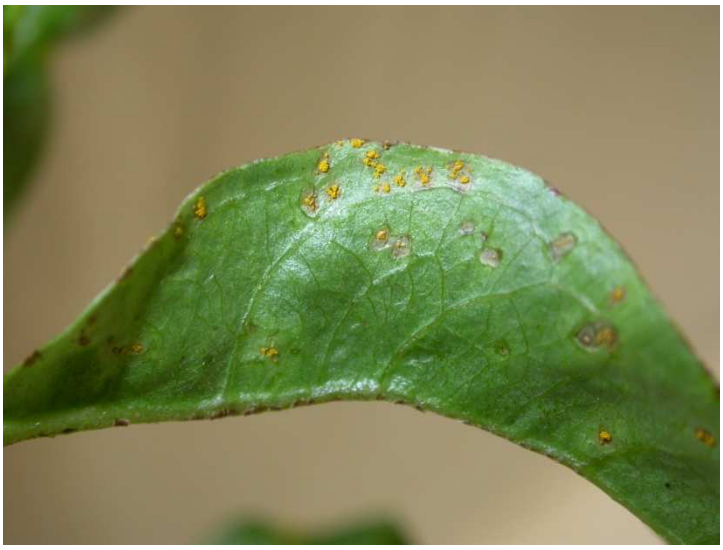

Eucalyptus obliqua

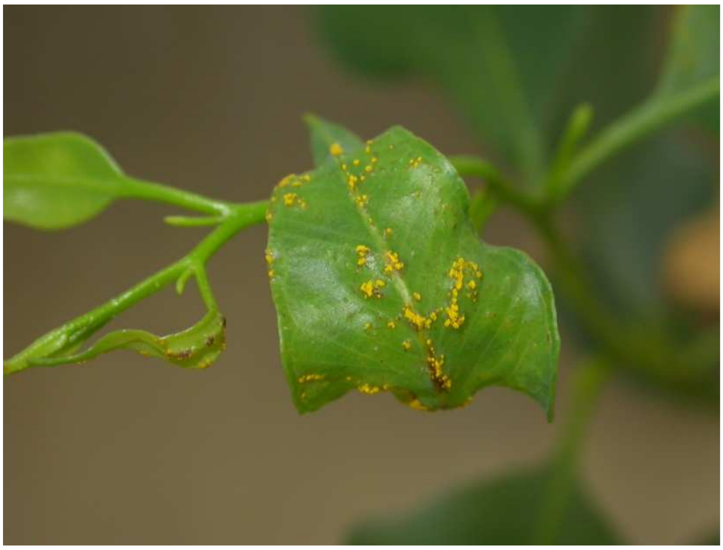

Eucalyptus occidentalis

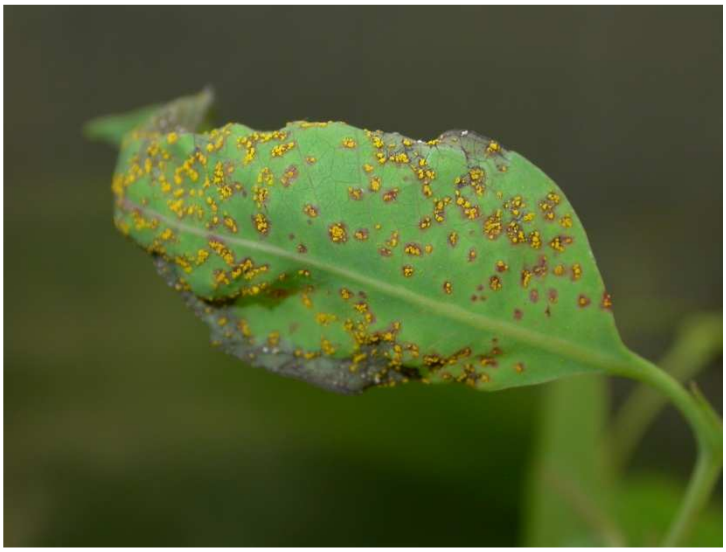

Eucalyptus olida

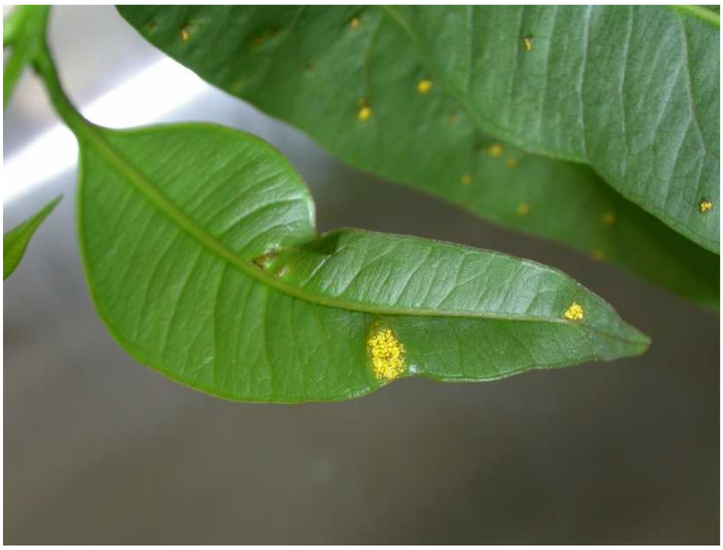

Eucalyptus pellita

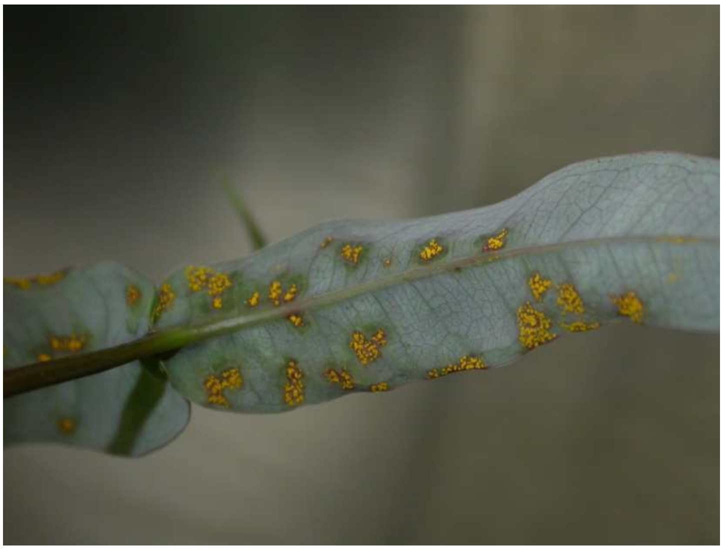

Eucalyptus pilularis

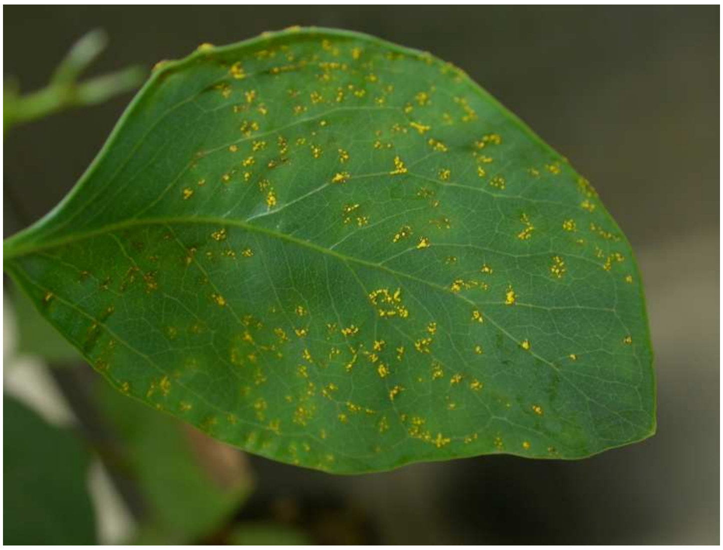

Eucalyptus populnea

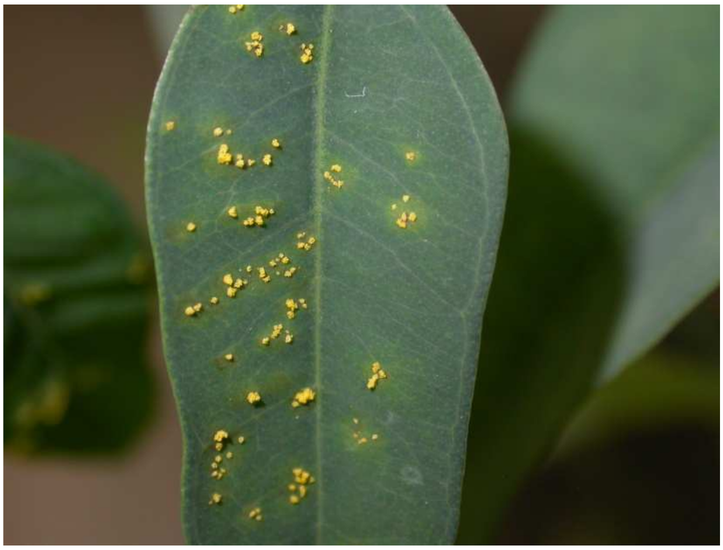

Eucalyptus punctata

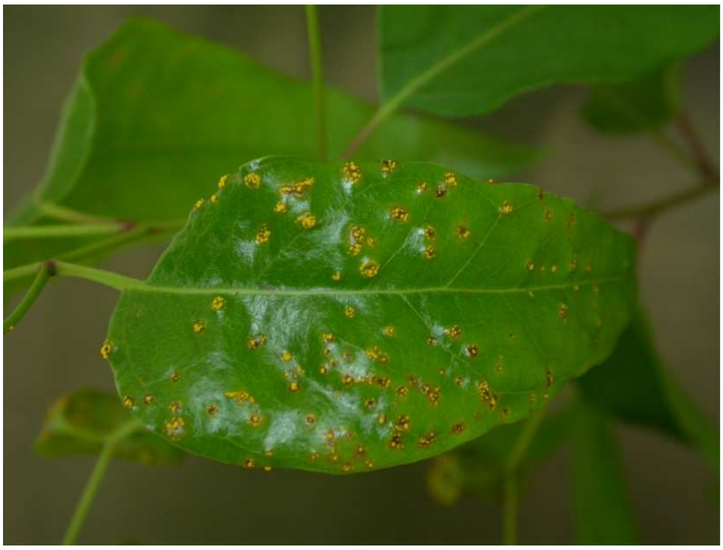

Eucalyptus regnans

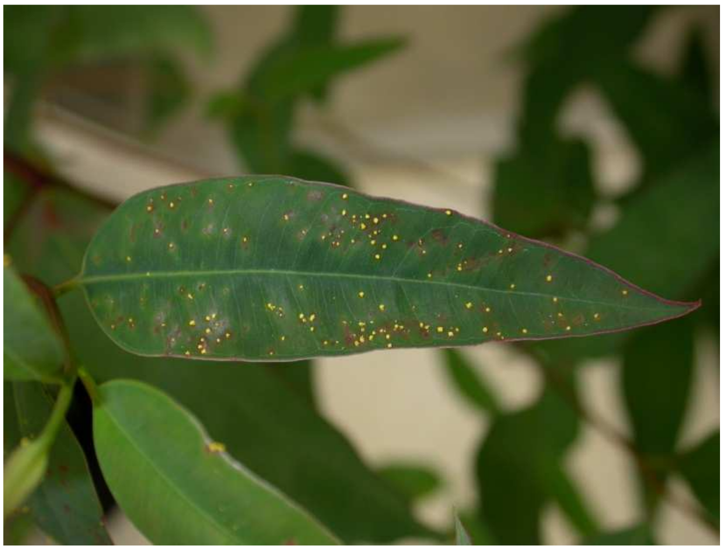
Eucalyptus resinifera subsp. *hemilampra*

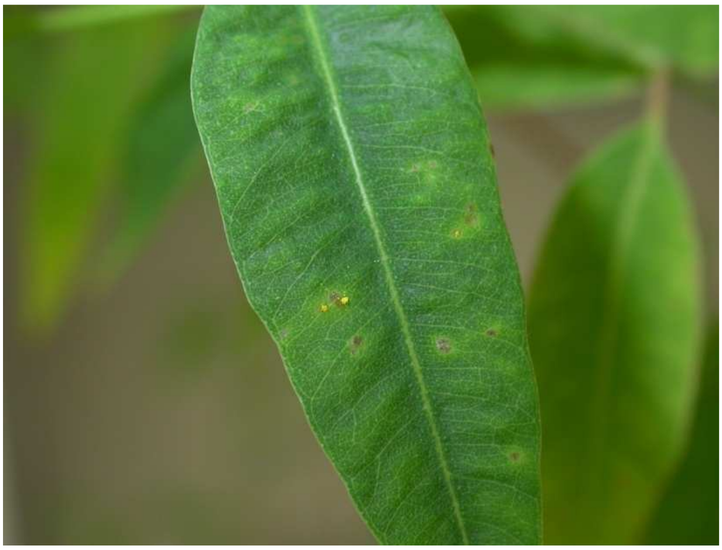
Eucalyptus saligna

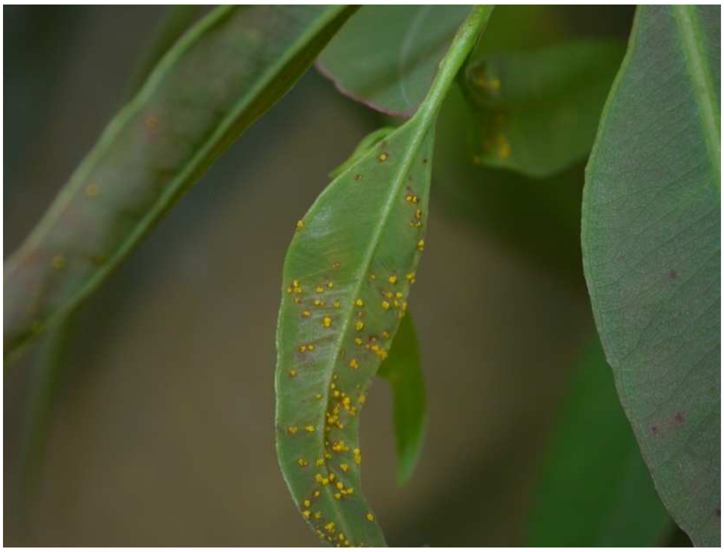
Eucalyptus siderophloia

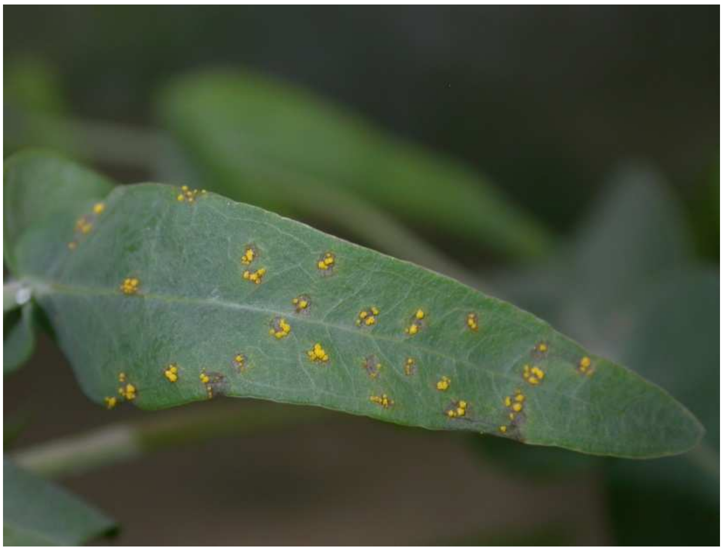
Eucalyptus smithii

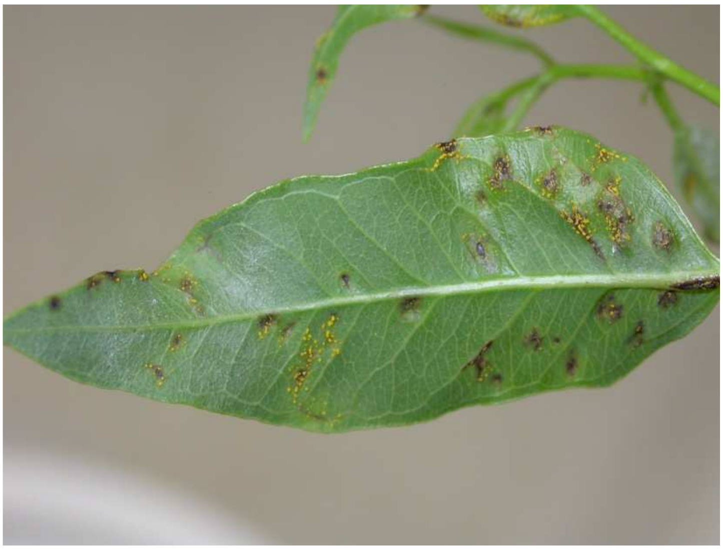
Eucalyptus tereticornis

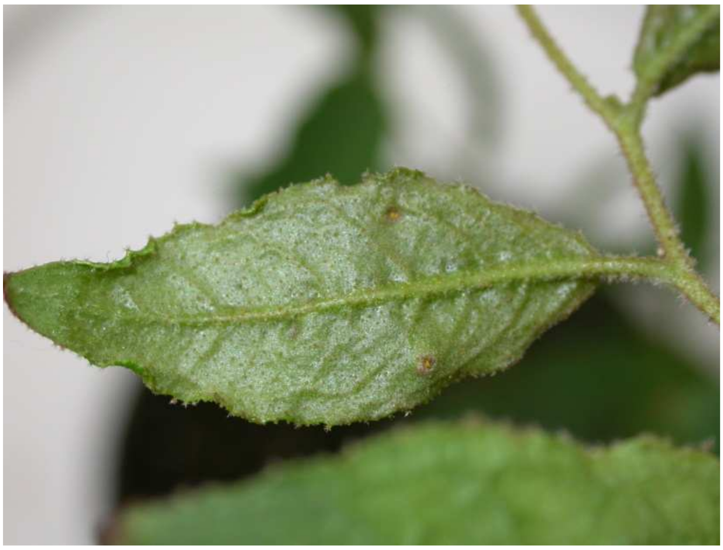
Eucalyptus tindaliae

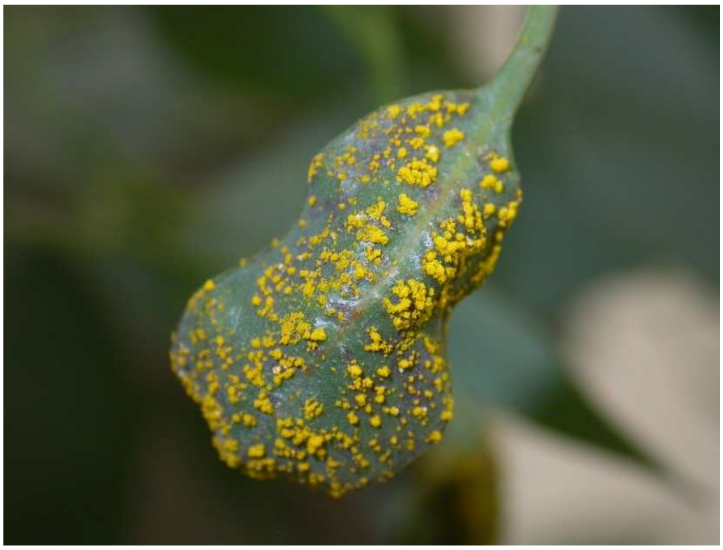
Eucalyptus wandoo subsp. *wandoo*

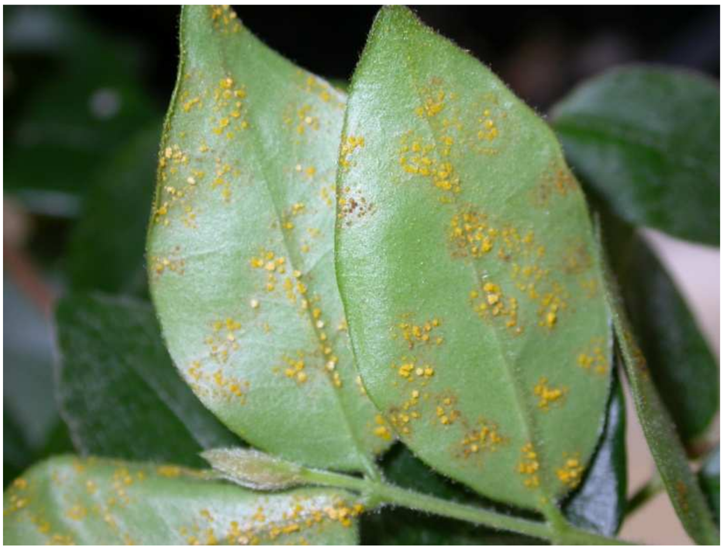
Gossia inophloia

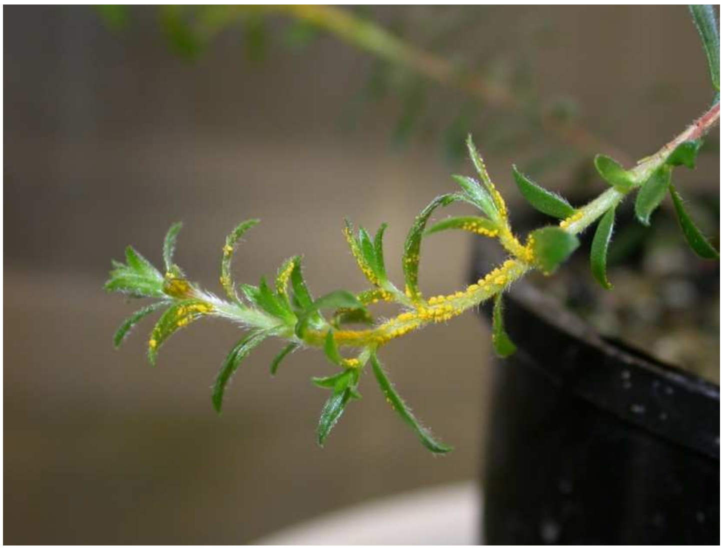
Kunzea ambigua hybrid

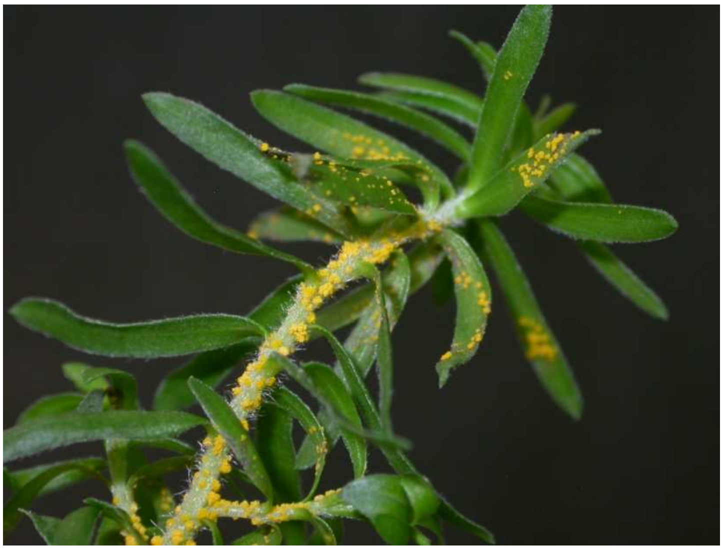
Kunzea baxteri

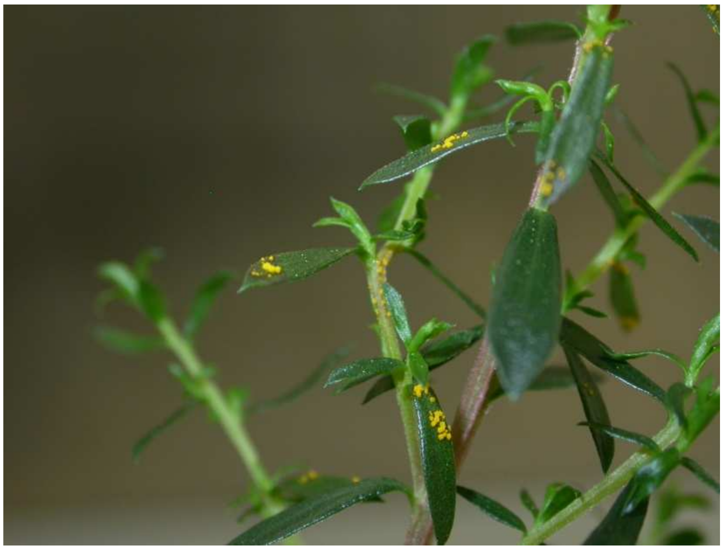
Kunzea ericoides

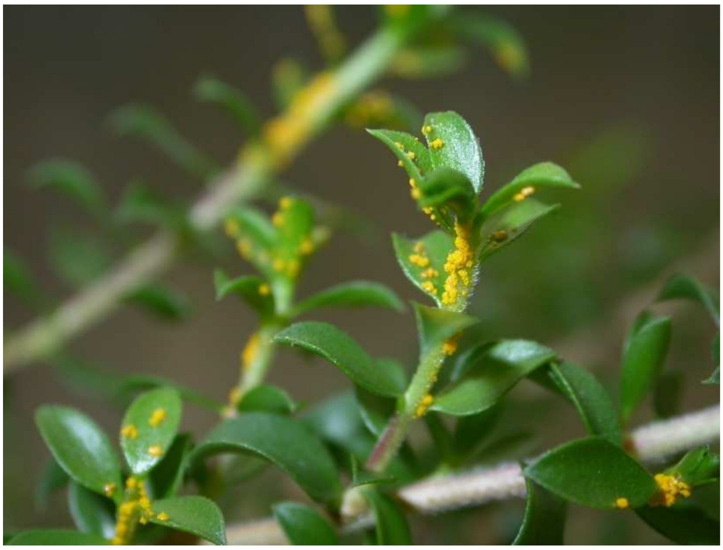
Kunzea pomifera

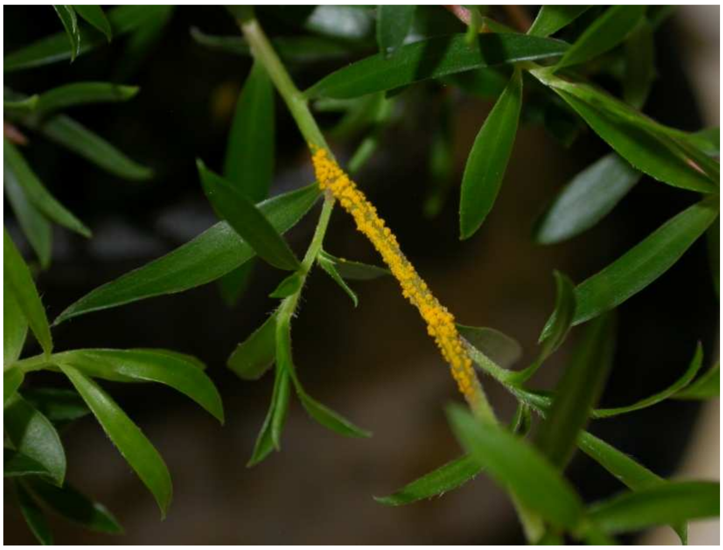

Leptospermum 'Day Dream'

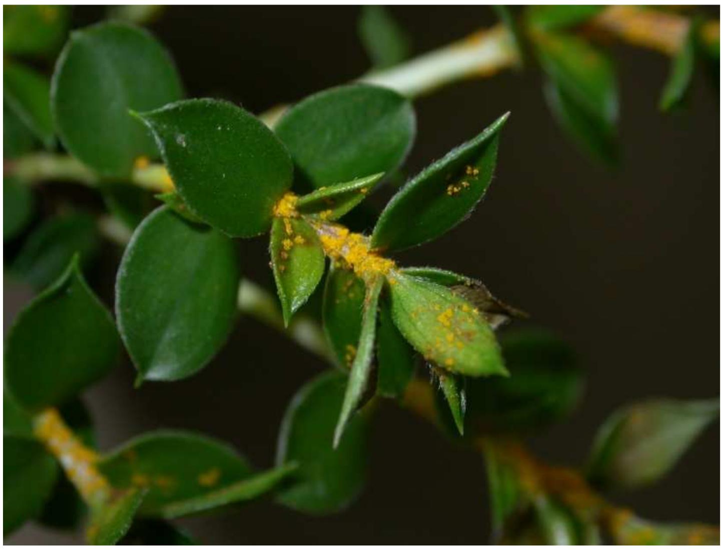

Leptospermum continentale 'Horizontalis'

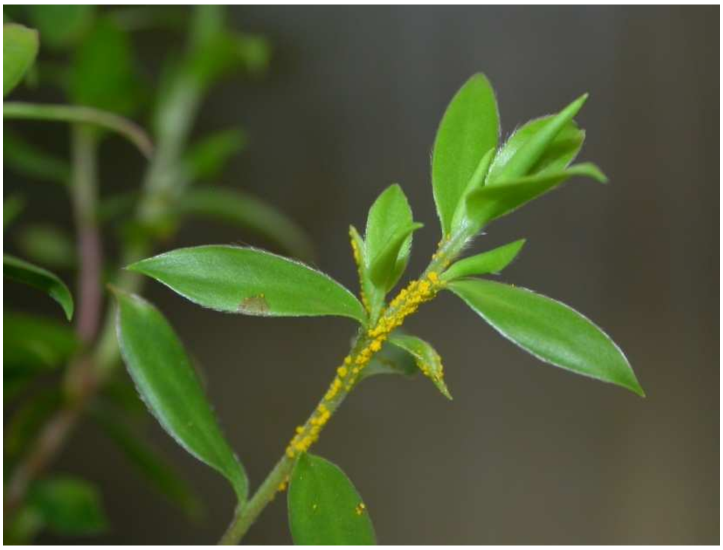

Leptospermum 'Lipstick'

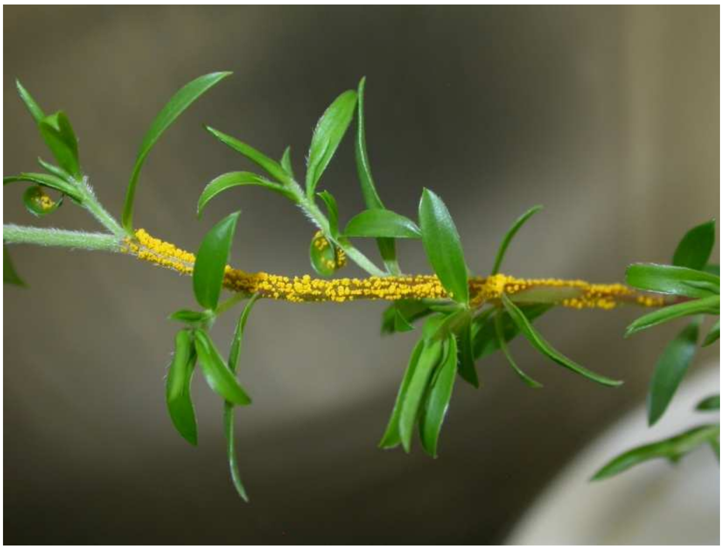

Leptospermum 'Love Affair'

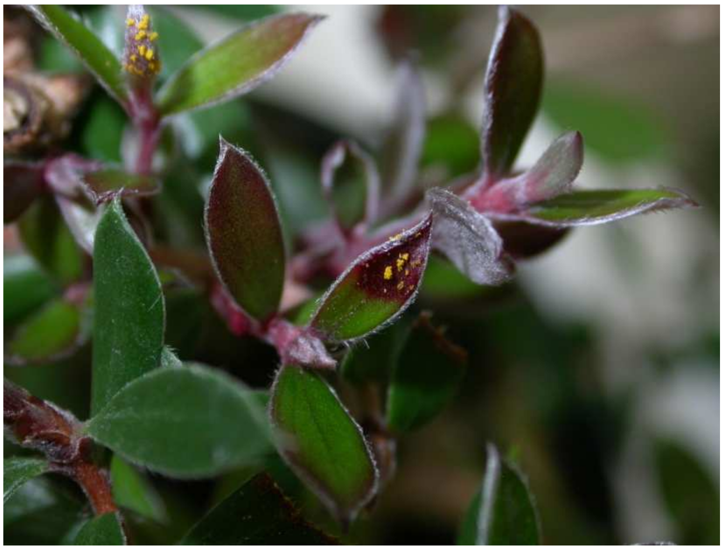

Leptospermum 'Mesmer Eyes'

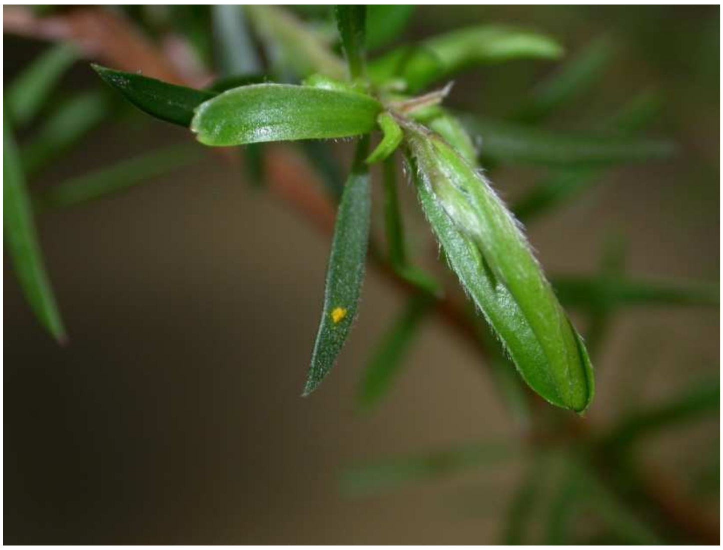

Leptospermum 'Pink Cascade'

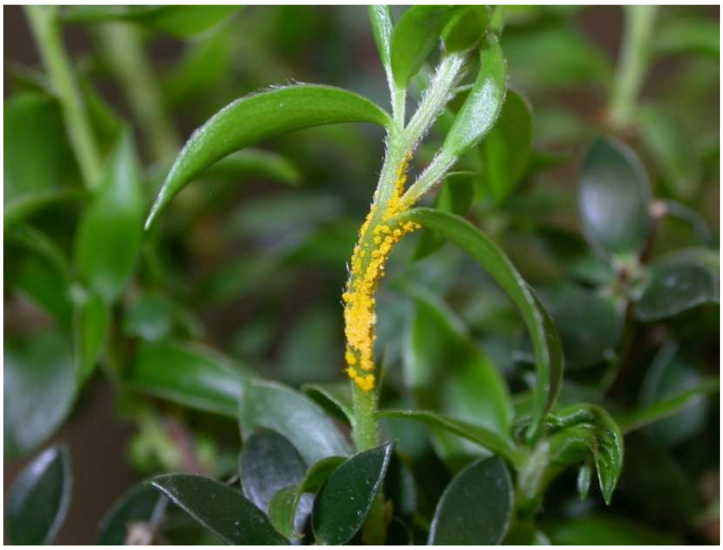
Leptospermum 'Rhiannon'

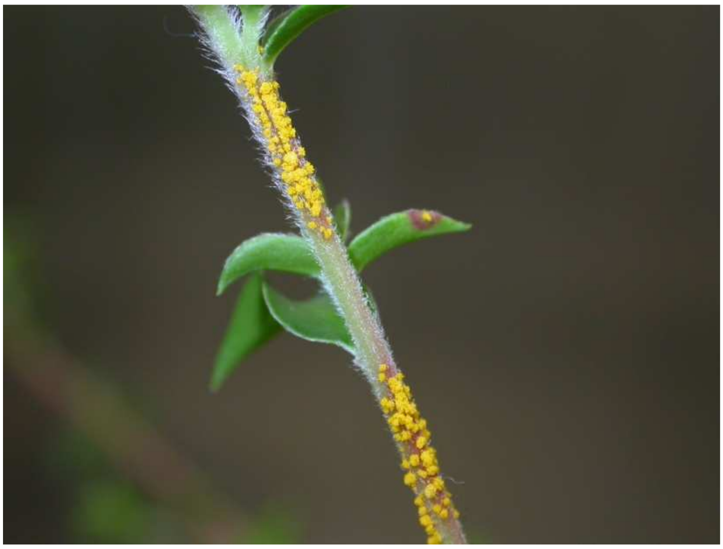
Leptospermum 'Riot'

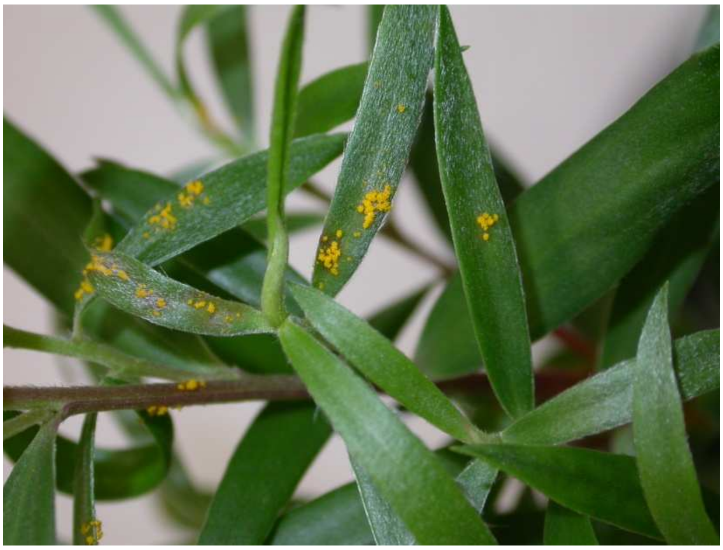
Leptospermum 'Rudolph'

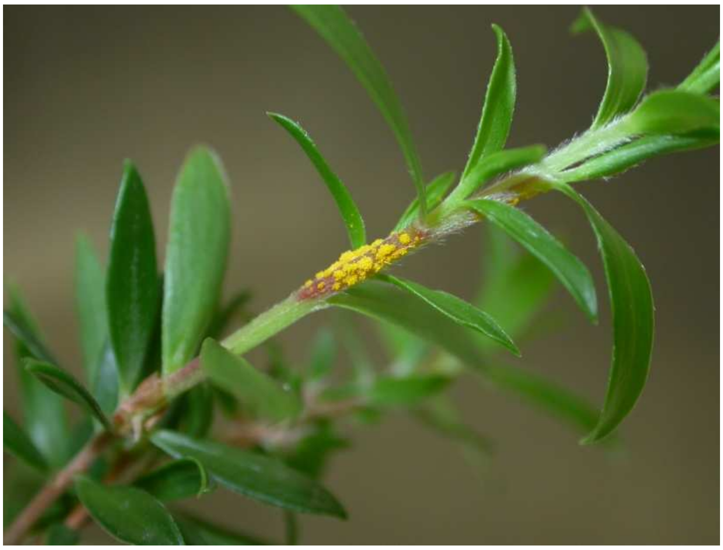
Leptospermum 'White Wave'

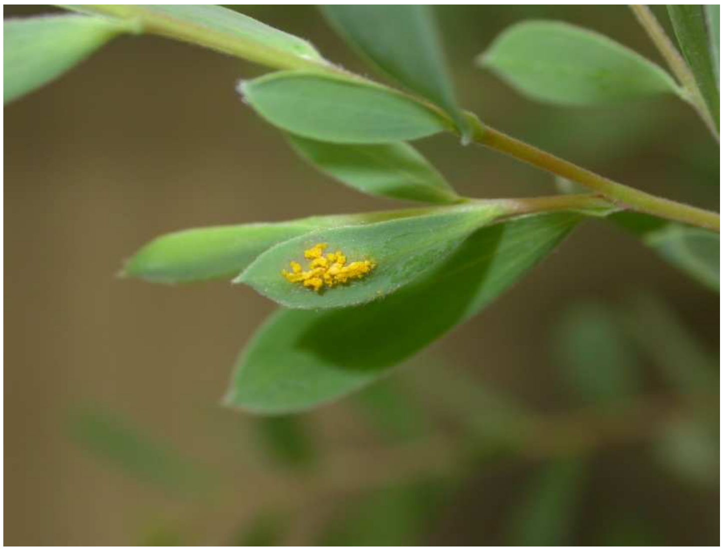
Leptospermum laevigatum

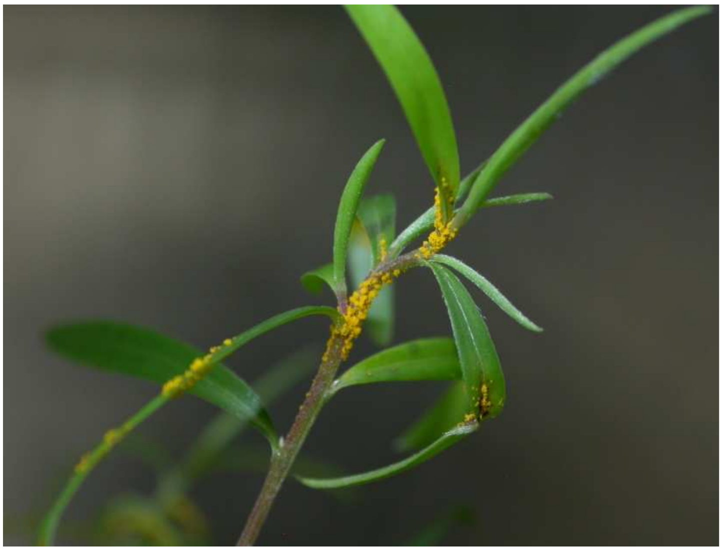
Leptospermum morrisonii 'Burgundy'

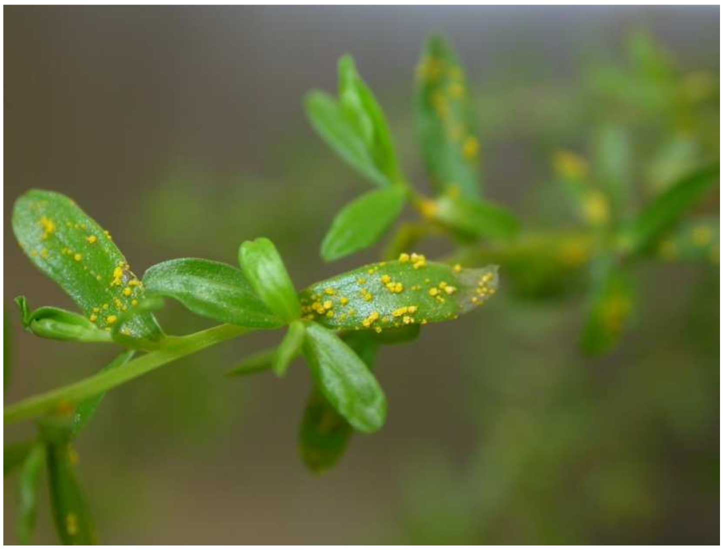

Leptospermum polygalifolium

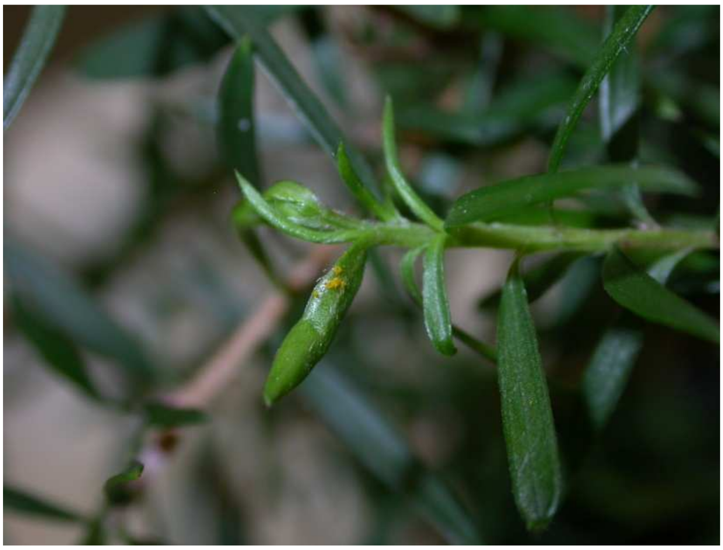

Leptospermum polygalifolium x scoparium

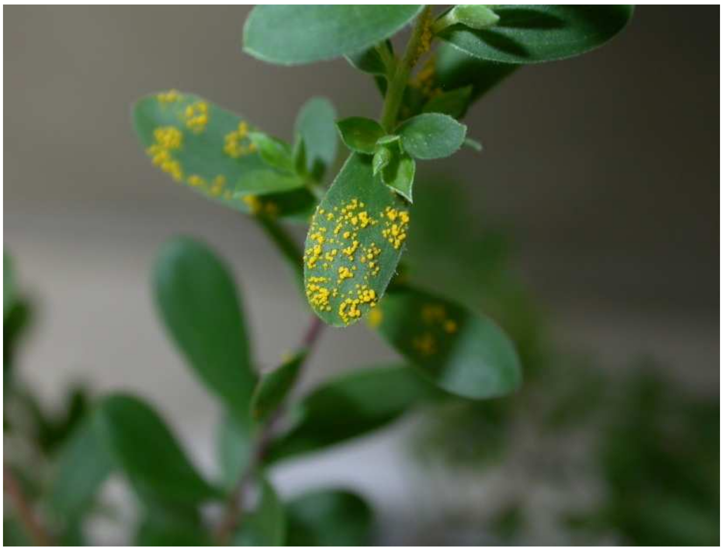

Leptospermum trinervium

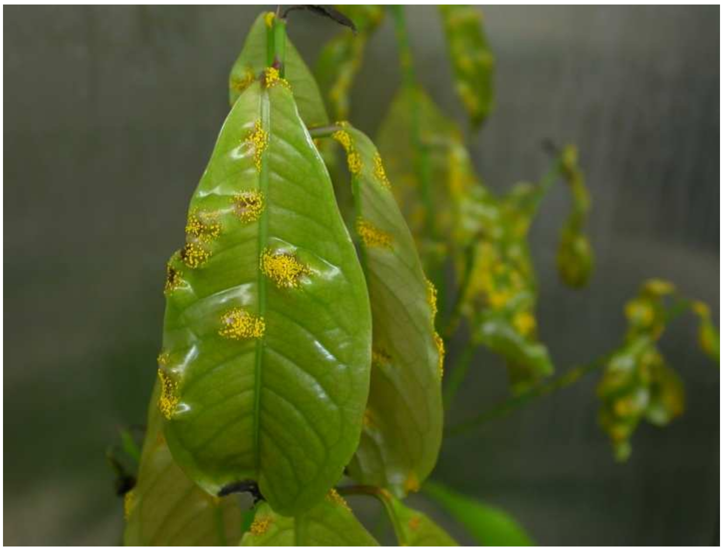

Lindsayomyrtus racemoides

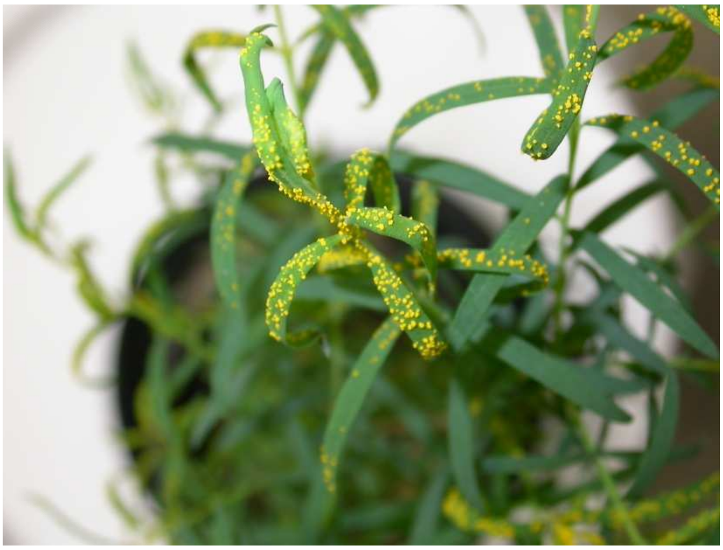

Melaleuca alternifolia

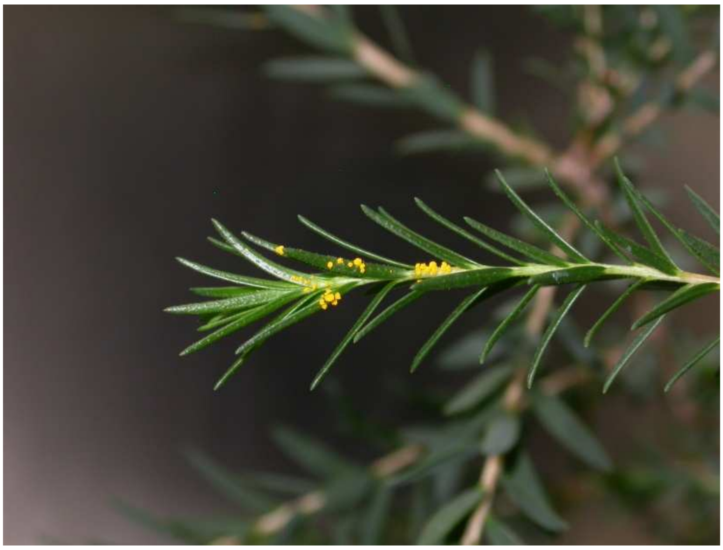

Melaleuca ericifolia

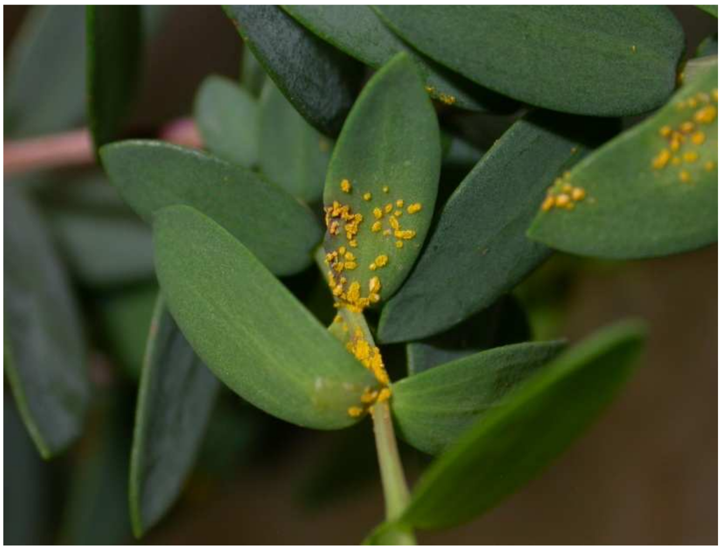

Melaleuca howeana

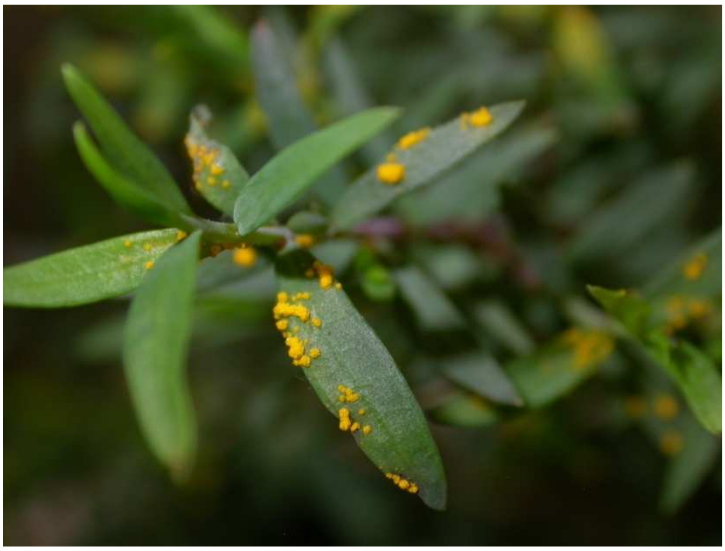

Melaleuca linariifolia

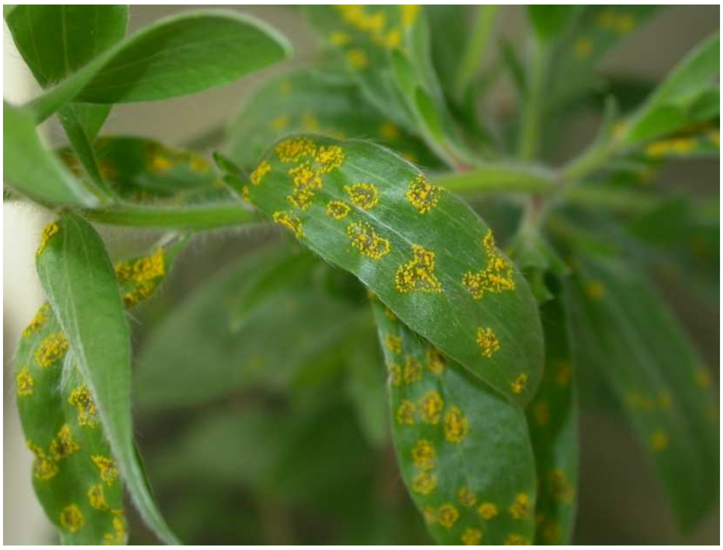

Melaleuca quinquenervia

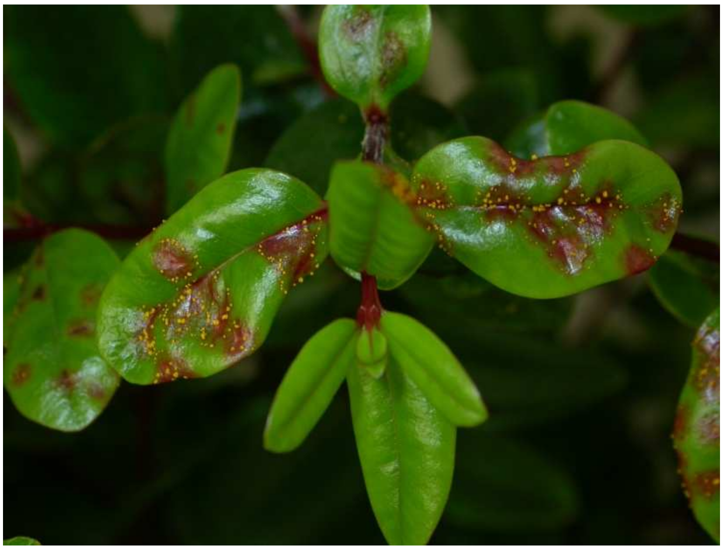

Metrosideros collina 'Tahiti'

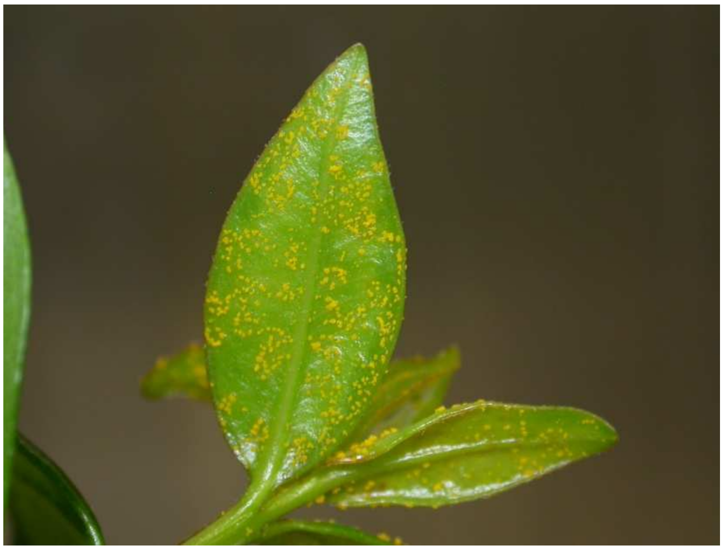

Metrosideros excelsa 'Golden Dawn'

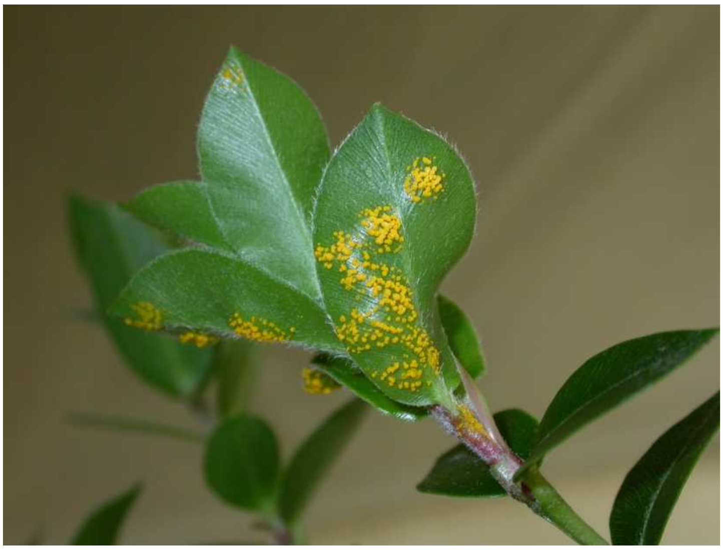

Metrosideros nervulosa

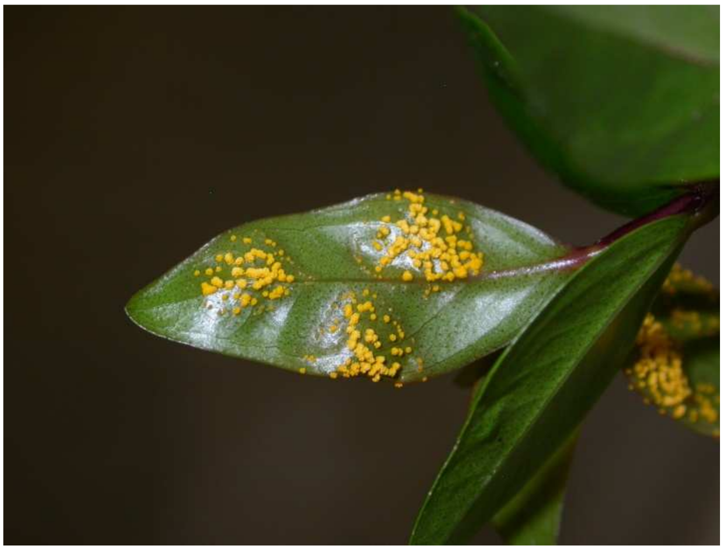
Metrosideros sclerocarpa

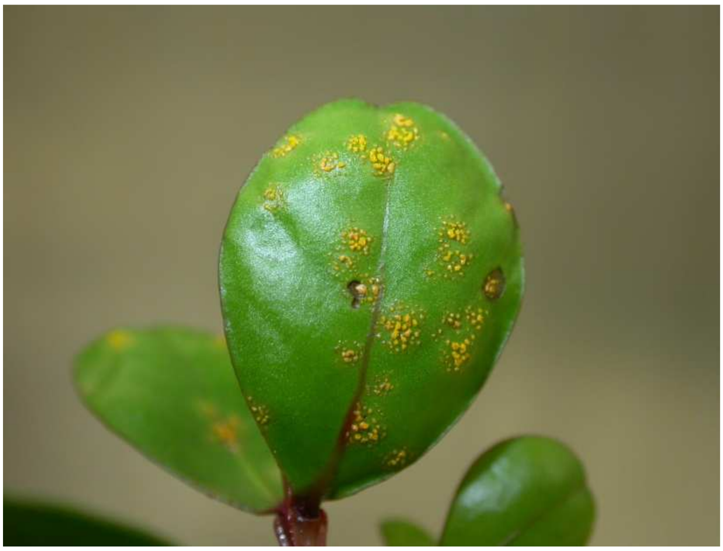
Osbornia octodonta

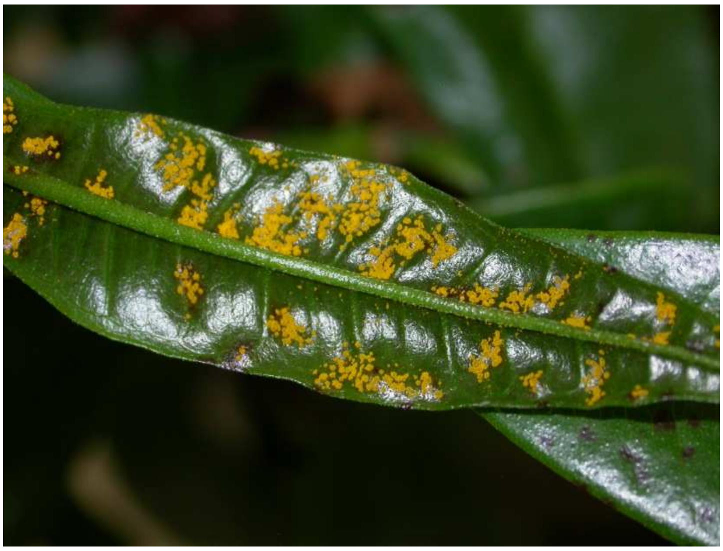
Pimenta dioica

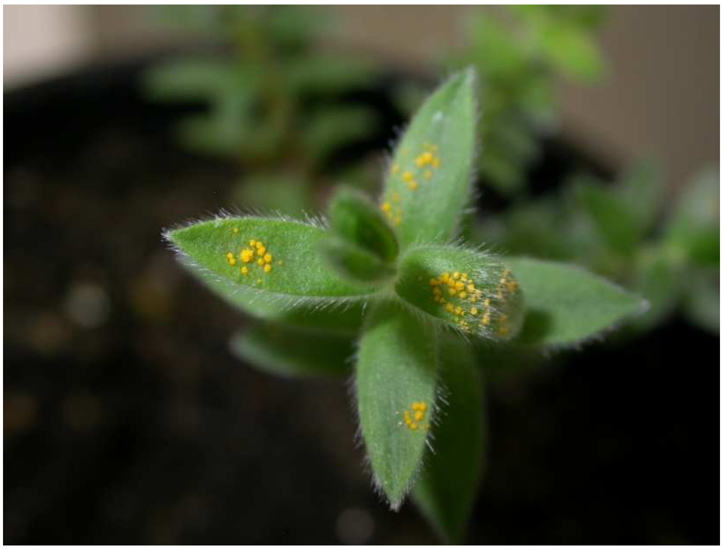
Regelia velutina

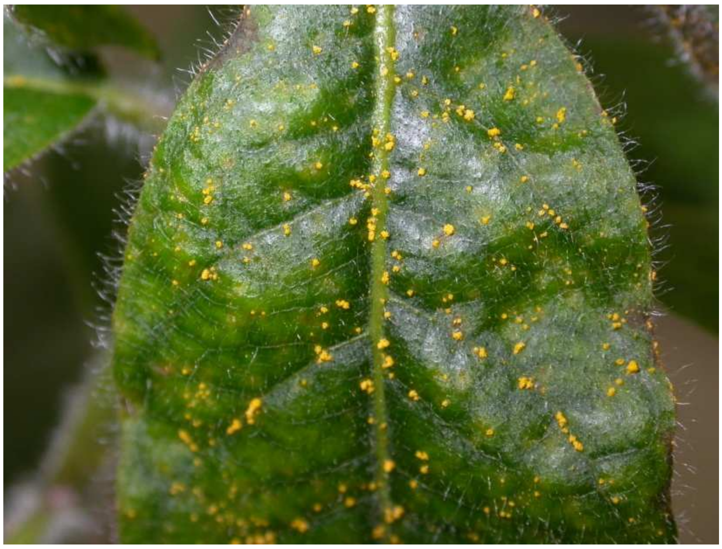
Syncarpia glomulifera

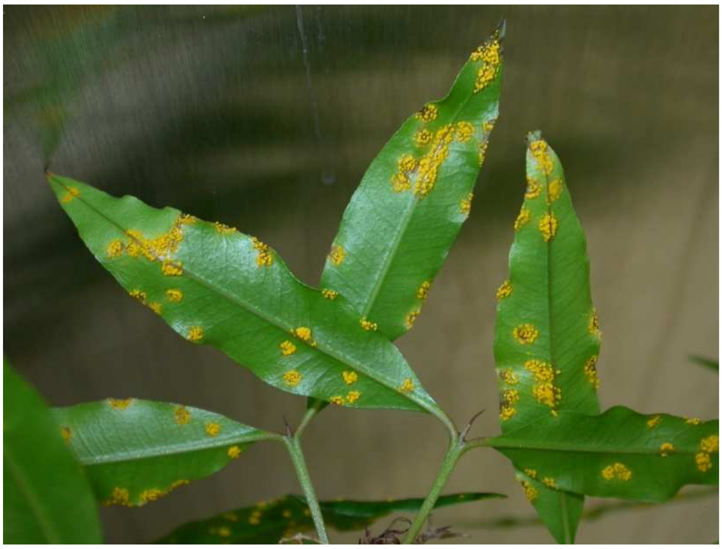
Syzygium anisatum

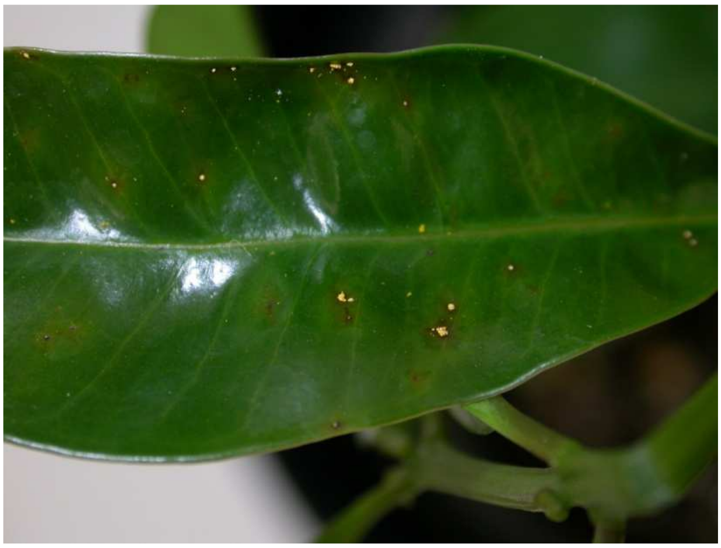

Syzygium australe 'Captain Cook'

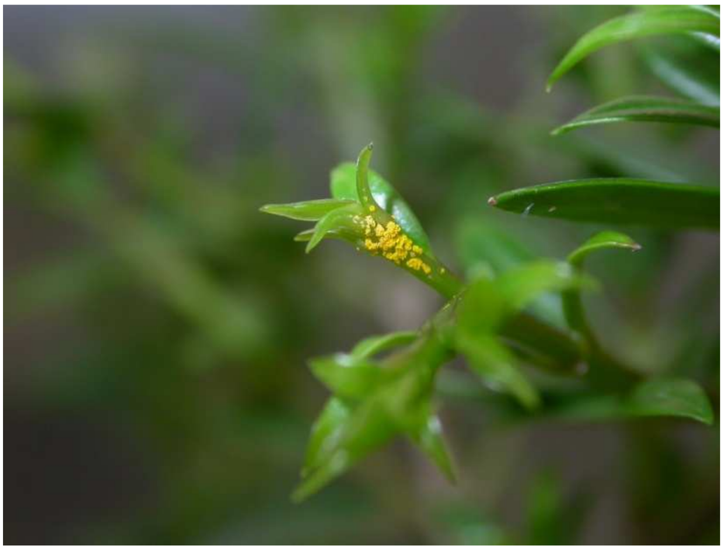

Syzygium australe ‘Meridian Midget'

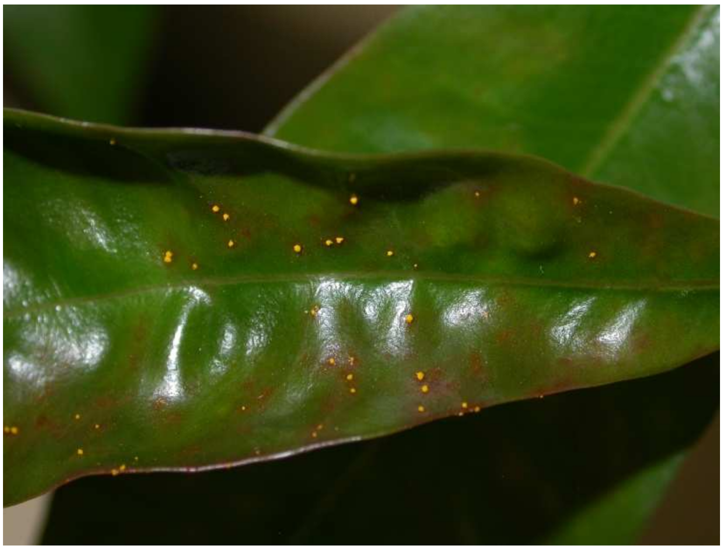

Syzygium fibrosum

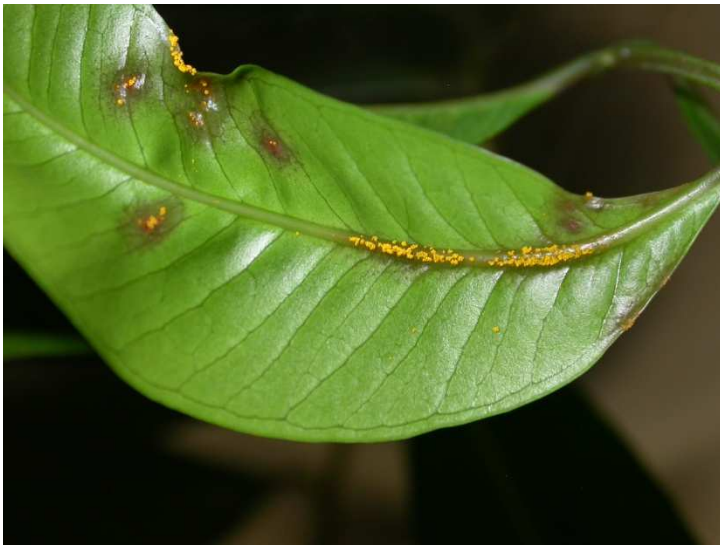

Syzygium floribundum

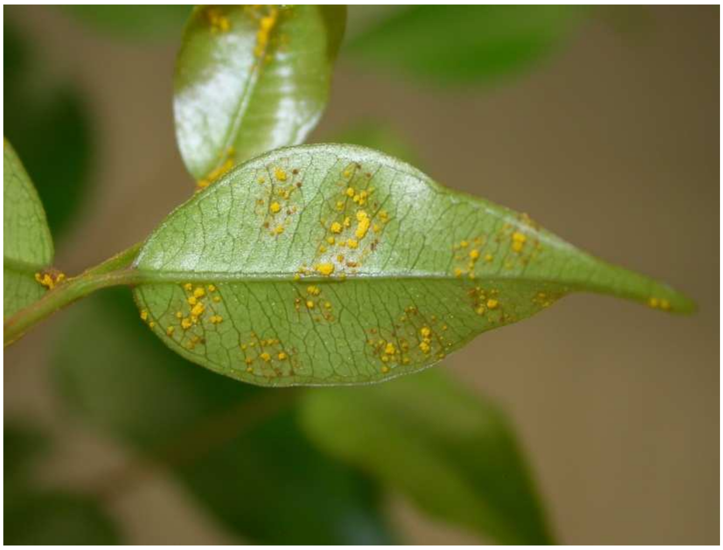

Syzygium francisii

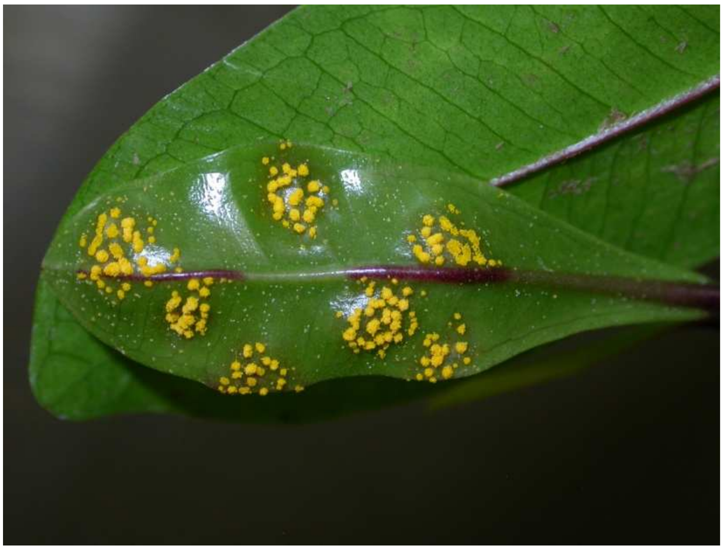

Syzygium fullagarii

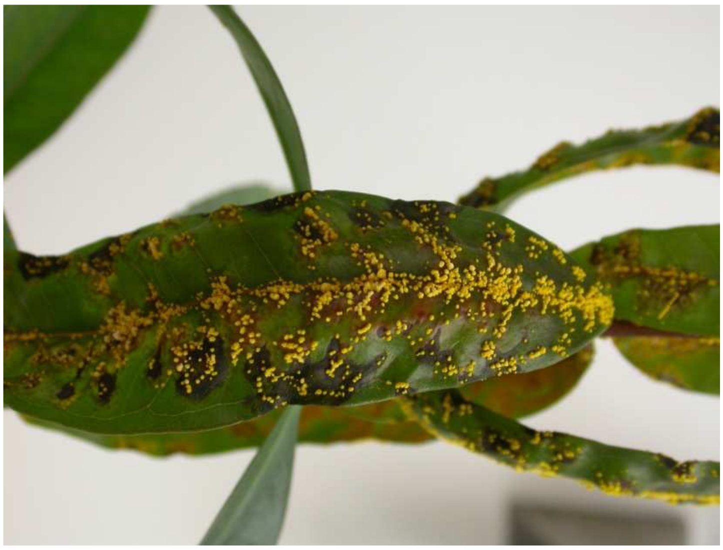

Syzygium jambos

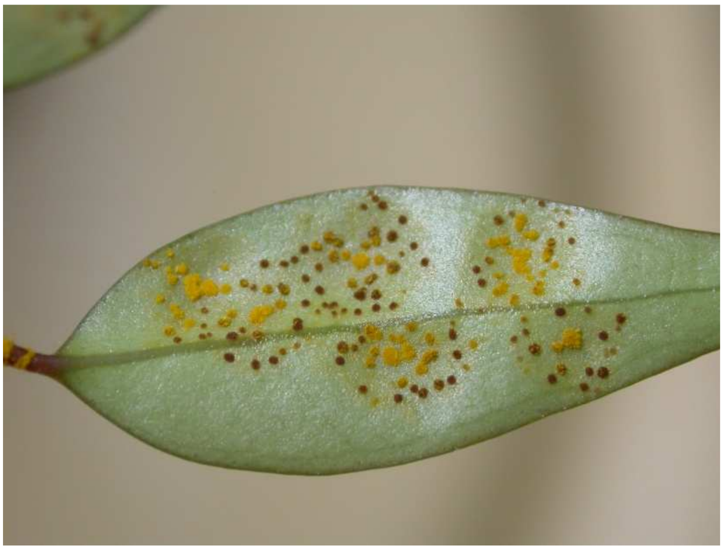

Syzygium luehmannii

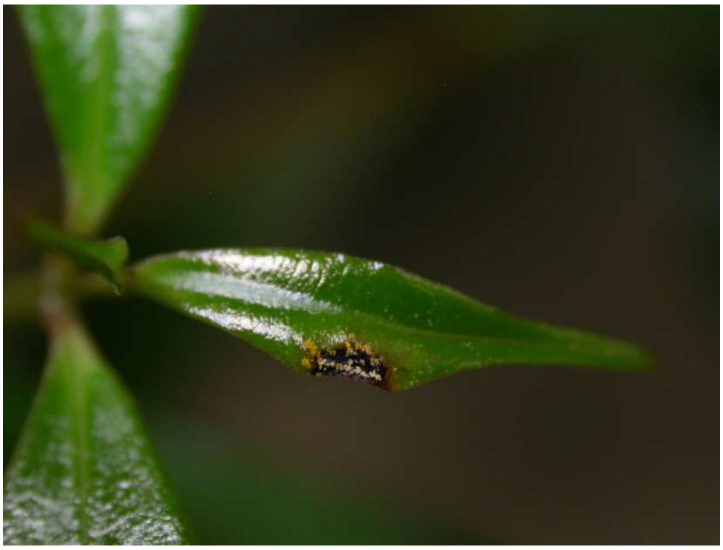

Syzygium oleosum

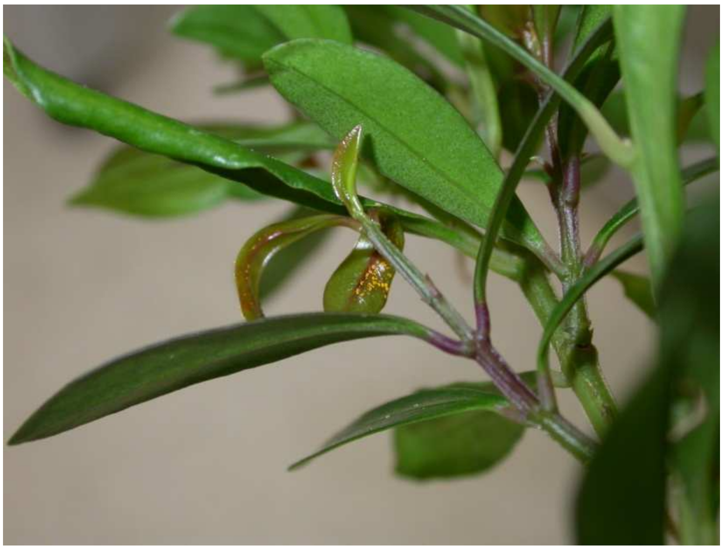

Syzygium smithii rheophytic form

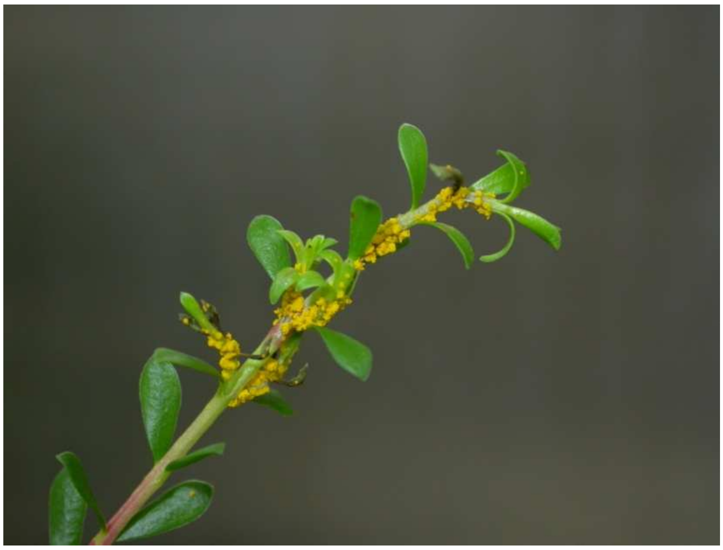

Thryptomene calycina

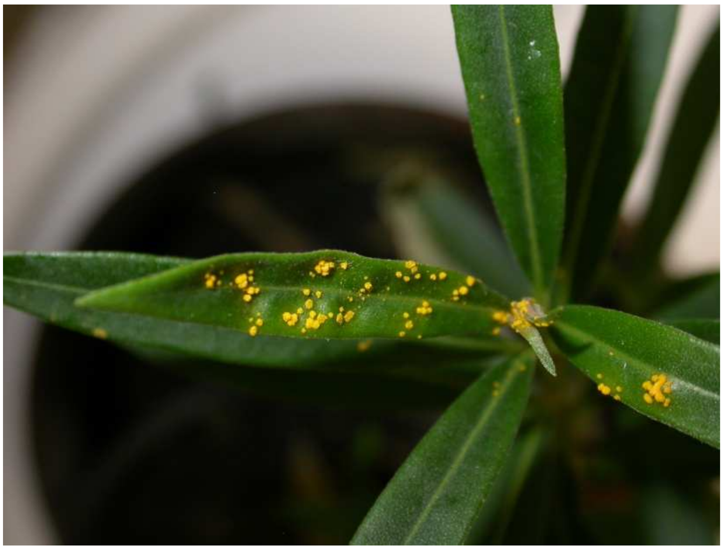

Tristania neriifolia

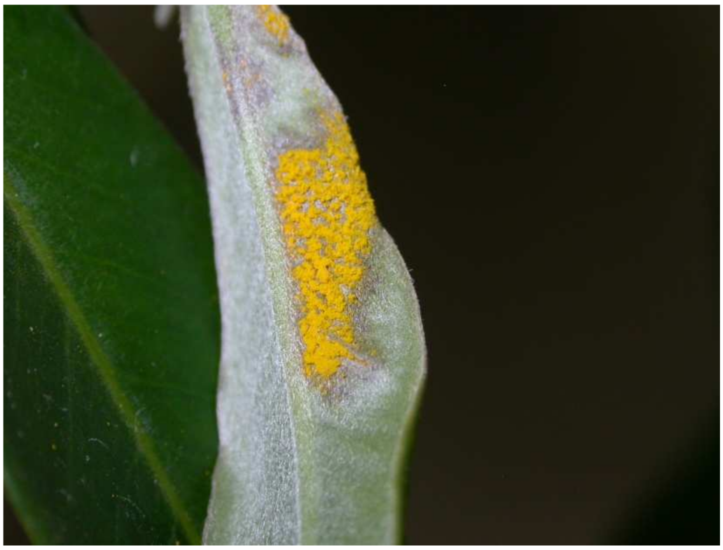

Tristaniopsis laurina

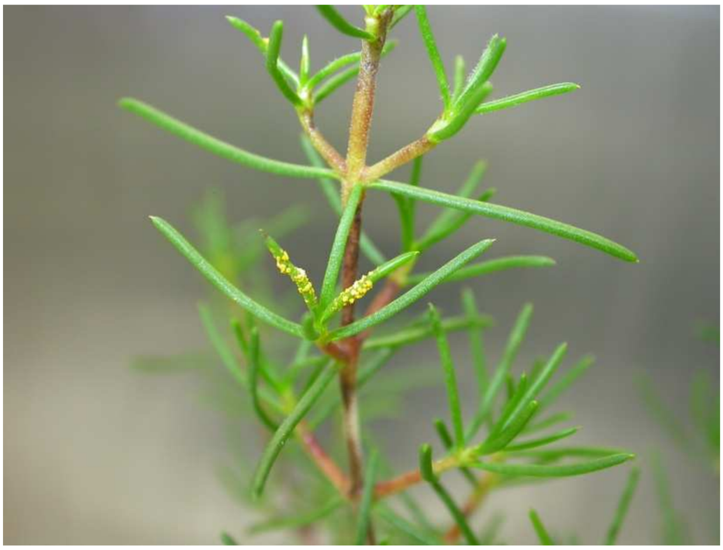

Verticordia chrysantha

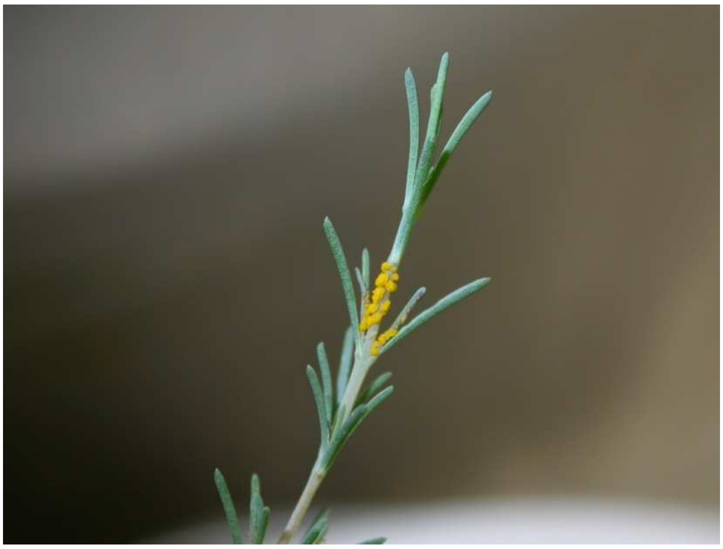

Verticordia plumosa

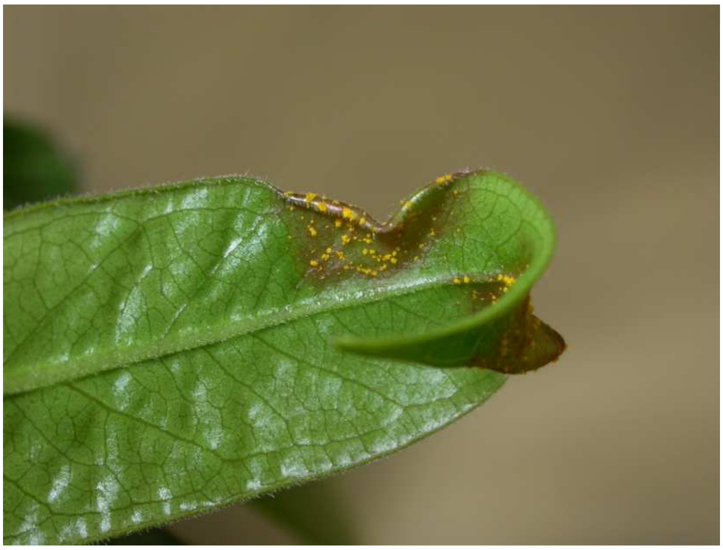

Xanthostemon chrysanthus
